# Breast Cancer Stem Cells with Tumor- versus Metastasis-Initiating Capacities Are Modulated by TGFBR1 Inhibition

**DOI:** 10.1016/j.stemcr.2019.05.026

**Published:** 2019-06-27

**Authors:** Flavia Fico, Mélanie Bousquenaud, Curzio Rüegg, Albert Santamaria-Martínez

**Affiliations:** 1 Tumor Ecology Lab, Department of Oncology, Microbiology and Immunology, Faculty of Science and Medicine, University of Fribourg, Fribourg, Switzerland; 2 Experimental and Translational Oncology Lab, Department of Oncology, Microbiology and Immunology, Faculty of Science and Medicine, University of Fribourg, Fribourg, Switzerland

**Keywords:** cancer stem cells, TGFBR1, breast cancer, metastasis

## Abstract

Cancer stem cells (CSCs) are defined by their ability to regenerate a tumor upon transplantation. However, it is not yet clear whether tumors contain a single CSC population or different subsets of cells with mixed capacities for initiating primary and secondary tumors. Using two different identification strategies, we studied the overlap between metastatic stem cells and tumor-initiating cells (TICs) in the MMTV-PyMT model. Our results show that in the MMTV-PyMT model, Lin^−^CD90^−^ALDH^high^ cells retained a high tumor-initiating potential (TIP) in orthotopic transplants, in contrast to Lin^−^CD24^+^CD90^+^, which retained higher metastatic capacity. Interestingly, suppression of TGFβ signaling increased TIC numbers. We here describe the existence of distinct populations of CSCs with differing capacities to initiate tumors in the primary or the secondary site. Inhibiting TGFβ signaling shifts the balance toward the former, which may have unanticipated implications for the therapeutic use of TGFβ/TGFBR1 inhibitors.

## Highlights

• Metastasis and tumor-initiating capacity are not necessarily shared by the same cell
• MMTV-PyMT CD90^−^ALDH^high^ tumor cells are lineage-restricted progenitors with TIC
• TGFBR1 inhibition prevents metastasis but drives the expansion of tumor-initiating cells

## Graphical Abstract

**Figure:**
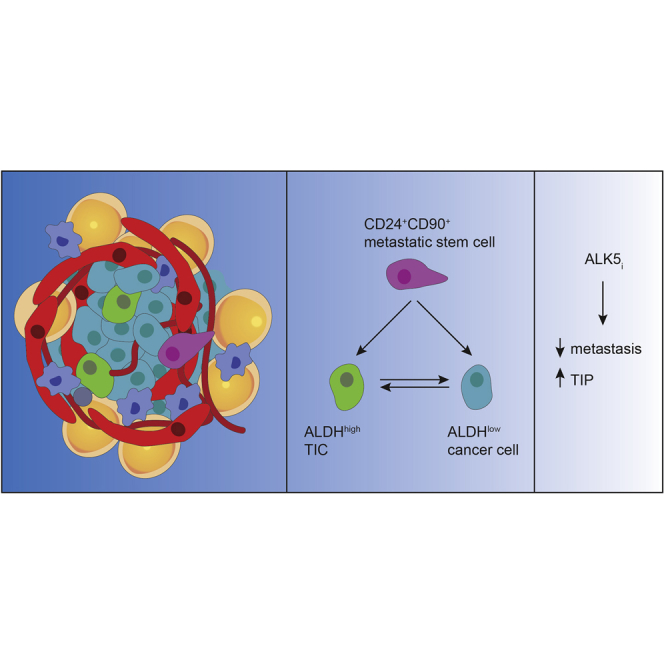

## Teaser

In this study, Santamaria-Martínez and colleagues isolated cancer stem cells from MMTV-PyMT mammary gland tumors based on the expression of either CD24 and CD90 or on ALDH activity and showed that metastasis- and tumor-initiating features are not necessarily shared by the same cell type. Additionally, they found that TGFBR1 inhibition prevents metastasis but promotes the expansion of tumor-initiating cells.

## Introduction

The cancer stem cell (CSC) hypothesis proposes that, similarly to what happens in normal tissues, heterogeneity within tumors is the consequence of their hierarchical organization, i.e., that many tumors are organized as a pyramid with CSCs at its apex. In many tumor types such as breast cancer, this subset of cells is known to sustain tumor growth but also, as we and others have shown, to lead metastatic colonization (Malanchi et al., 2012). The latter is particularly important since over 90% of cancer-related deaths are due to metastatic disease. Experimentally, CSCs are defined as tumor-derived cells that have the exclusive ability to regenerate a tumor upon transplantation—with all its full complexity and heterogeneity. To assess this capacity, typically CSCs are purified from tumors and tested for their tumor-initiating ability in limiting dilution assays. However, the lack of universal CSC markers poses a serious problem in understanding how homogeneous the CSC pool is.

A few years ago, Weinberg's lab proposed that CSCs can be generated from the epithelial-to-mesenchymal transition (EMT) (Mani et al., 2008), a process that confers motility and invasiveness to cancer cells and therefore is advantageous to metastasis. However, most secondary tumors derived from carcinomas show an epithelial morphology resembling that of the primary tumor. This suggests that metastatic cells may need to undergo a reverse process, the mesenchymal-to-epithelial transition, once they have colonized a secondary organ (Celia-Terrassa et al., 2012, Ocana et al., 2012, Tsai et al., 2012). Interestingly, the use of different isolation strategies in the same model allowed some researchers to distinguish between two subsets of CSCs according to their mesenchymal features: EMT-CSCs and MET-CSCs (Liu et al., 2014). Nevertheless, it is not clear whether CSC transition from one state to the other or two different CSC subpopulations exist in a tumor at a given time. Therefore, understanding whether tumor-initiating cells (TICs) are metastatic is essential in the design of rational targeted therapies and more accurate CSC isolation protocols. Since both tumor-initiating and metastatic CSCs need a different set of features to initiate tumors, we aimed at studying whether they are the same cell population. Here, we use the MMTV-PyMT model to identify two subgroups of CSCs and show that transforming growth factor β (TGFβ) receptor 1 (TGFBR1)/ALK5 inhibition prevents metastasis but not tumor initiation.

## Results

### CD90^−^ALDH^high^ Cells Are Lineage-Committed CSCs

**Figure 1:**
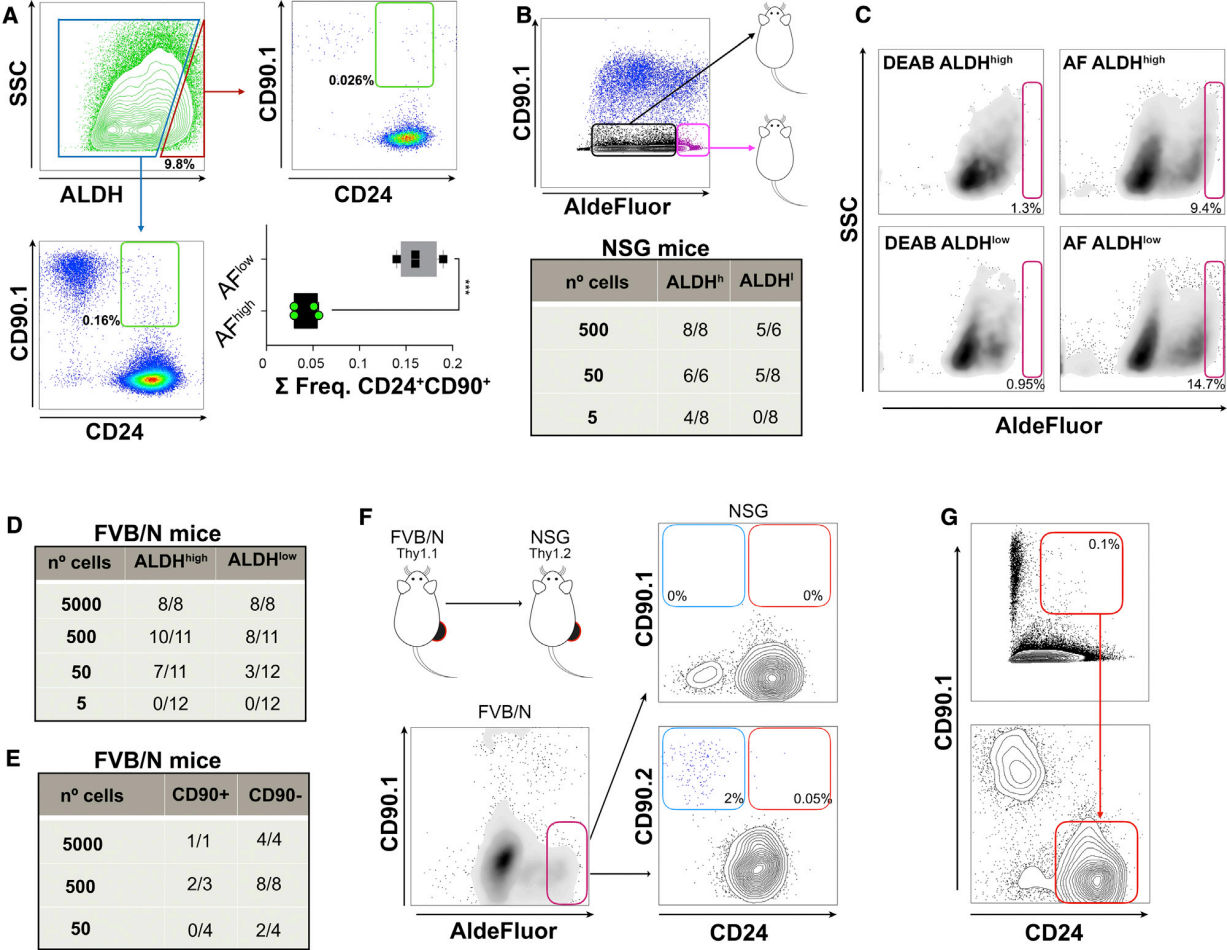
Metastatic Stem Cells versus Tumor-Initiating Cells (A) FACS analyses of MMTV-PyMT fresh tumors. Lin^−^ALDH^high^ and Lin^−^ALDH^low^ DAPI-negative singlets were gated and analyzed for the expression of CD24 and CD90 (absolute frequency, n = 4 independent tumors, paired Student's t test). (B) MMTV-PyMT cells from fresh tumors were FACS sorted using the AldeFluor assay, then counted and injected orthotopically in limiting dilution assays in NSG mice. The presence or absence of tumors was evaluated for a maximum of 3 months after injection. Data were analyzed using ELDA Extreme. (C) Cytograms showing the AldeFluor assay profiles of tumors derived from Lin^−^CD90^−^ALDH^high^ and Lin^−^CD90^−^ALDH^low^ cells. (D) MMTV-PyMT cells from fresh tumors were FACS sorted using the AldeFluor assay, then counted and injected orthotopically in limiting dilution assays in FVB/N mice. The presence or absence of tumors was evaluated for a maximum of 3 months after injection. Data were analyzed using ELDA Extreme. (E) MMTV-PyMT cells from fresh tumors were FACS sorted for CD24CD90, then counted and injected orthotopically in limiting dilution assays in FVB/N mice. The presence or absence of tumors was evaluated for a maximum of 3 months after injection. Data were analyzed using ELDA Extreme. (F and G) Lin^−^CD90^−^ALDH^high^ cells from MMTV-PyMT spontaneous tumors (FVB/N, Thy1.1) were FACS sorted and transplanted into NSG (Thy1.2) mice to determine lineage restrictions (F). Note that host-derived cancer-associated fibroblasts are CD90.2. (G) FACS-sorted Lin^−^CD24^+^CD90^+^ cells from MMTV-PyMT spontaneous tumors (upper cytogram) can give rise to tumors with Lin^−^CD24^+^CD90^−^ cells when transplanted syngeneically (lower cytogram).

The ability of cancer cells to form metastasis in the lungs is typically tested in intravenous injections in the tail vein. Using the MMTV-PyMT model, we have previously shown that most of this capacity is retained by Lin^−^CD24^+^CD90^+^ cells (Malanchi et al., 2012). However, what is not clear is the extent of the overlap, if any, between metastatic stem cells and primary TICs. The AldeFluor assay, which determines aldehyde dehydrogenase (ALDH) activity, is frequently used to identify cells that possess higher tumor-initiation capacity (Wan et al., 2014). We therefore performed fluorescence-activated cell sorting (FACS) analyses on PyMT cells, which revealed that there is no significant overlap between Lin^−^CD24^+^CD90^+^ cells and Lin^−^ALDH^high^ cells, suggesting that both strategies identify different populations (Figure 1A). To uncouple the effects on primary tumor- and metastasis-initiation capacity, we next performed orthotopic transplantation of CD90-depleted cancer cells in limiting dilution assays. Our results show that Lin^−^CD90^−^ALDH^high^ cells possessed 20.7-fold higher tumor-initiating potential (TIP) than Lin^−^CD90^−^ALDH^low^ (p < 0.0001), with an estimated stem cell frequency of 1 in 7 cells when injected in NSG mice (Figure 1B). This indicates that Lin^−^CD90^−^ALDH^high^ is a population enriched in bona fide CSCs with TIP. It is worth noting that in those Lin^−^CD90^−^ALDH^low^ tumors that are able to grow, ALDH activity is recovered (Figures 1C, S1A, and S1B), which suggests that a subset of Lin^−^ALDH^low^ cells can replenish the Lin^−^ALDH^high^ fraction. The CSC assay evaluating TIP has been critically regarded as a means to test the ability of tumor cells to evade or modulate the immune system, particularly T cell- and natural killer (NK) cell-mediated killing (Quintana et al., 2008). Therefore, to avoid potential confounding effects of the lack of an immune system, we performed the same assay in FVB/N mice. In fully immunocompetent mice, Lin^−^CD90^−^ALDH^high^ cells still have higher TIP (p < 0.03), although this is reduced to 2.7-fold compared with Lin^−^CD90^−^ALDH^low^ cells (Figure 1D). Furthermore, the difference in TIP compared with NSG grafts is reduced by 16.8-fold and 2.2-fold in Lin^−^CD90^−^ALDH^high^ and Lin^−^CD90^−^ALDH^low^, respectively (Figure S1C). Next, we compared the tumor-initiation capacity of Lin^−^CD24^+^CD90^+^ and Lin^−^CD24^+^CD90^−^ cells. Our results show that Lin^−^CD24^+^CD90^+^ cells have a decreased tumor-initiating ability in orthotopic limiting dilution assays (p < 0.02, Figures 1E and S1C). To test whether CD90^−^ cells can give rise to CD90^+^ cells, we took advantage of the allelic difference in CD90 between FVB/N (CD90.1) and NSG (CD90.2) mouse strains and were able to confirm that neither Lin^−^ALDH^high^CD90^−^ nor Lin^−^ALDH^low^CD90^−^ cells are able to give rise to Lin^−^CD24^+^CD90^+^ *in vivo* (Figure 1F). However, CD90^+^ tumors can give rise to CD90^−^ cells (Figure 1G). As expected, CD90-depleted tumors show a 19-fold lower metastatic index when compared with CD90-containing tumors (Figure S1D). These results indicate that in the MMTV-PyMT model, CD90^−^ tumor cells are lineage restricted but they harbor a strong TIP, while CD90^+^ cancer cells retain a high metastatic potential.

### CSC Populations Differ in Their Mesenchymal Traits

**Figure 2:**
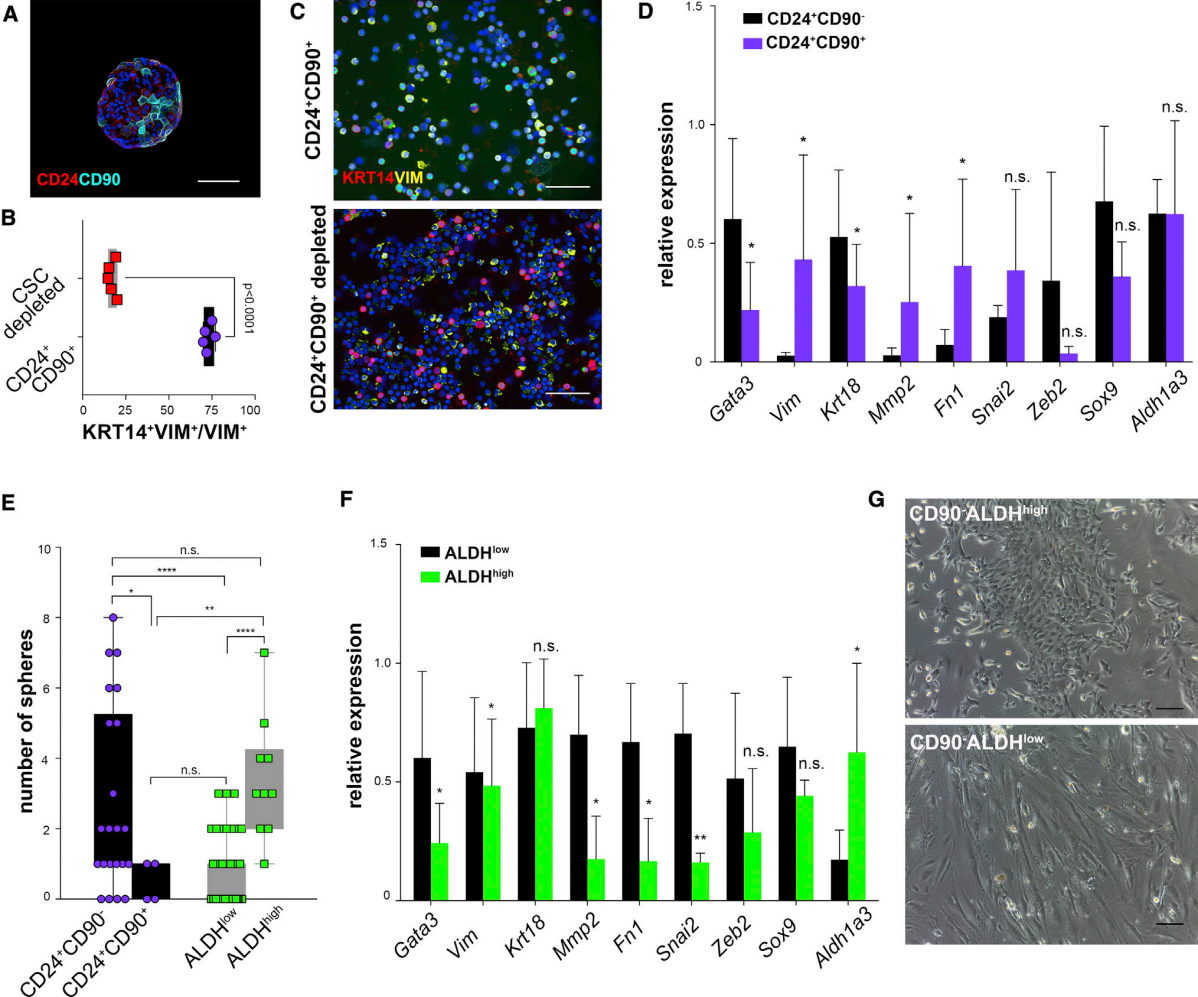
Characterization of CSCs (A) Immunofluorescent analysis of mammospheres from the MMTV-PyMT model revealed that they contain a small fraction of CD24^+^CD90^+^ cells. Scale bar, 100 μm. (B and C) FACS-sorted Lin^−^CD24^+^CD90^+^ and Lin^−^CD24^+^CD90^+^-depleted cells (B) were cytospinned, fixed, and stained for cytokeratin 14 and vimentin (C; scale bars, 100 μm). The number of double-positive cells in vimentin^+^ cells was calculated using unpaired Student's t test (n = 5). (D) qPCR on FACS-sorted Lin^−^CD24^+^CD90^+^ and Lin^−^CD24^+^CD90^−^ cells from fresh MMTV-PyMT tumors showed differences in gene expression (n = 6 independent tumors; *Rplp0* was used as a housekeeping gene; paired Student's t test). (E) FACS sorting and culture of different populations of cells revealed that most of the sphere-formation ability is retained by ALDH^high^ cells (n = 22 CD24^+^CD90^−^, n = 4 CD24^+^CD90^+^, n = 49 ALDH^low^, n = 10 ALDH^high^, for three independent tumors, one-way ANOVA and Fisher's LSD). (F) qPCR on FACS-sorted Lin^−^CD90^−^ALDH^high^ and Lin^−^CD90^−^ALDH^low^ cells from fresh MMTV-PyMT tumors showed differences in gene expression (n = 4 independent tumors; *Rplp0* was used as a housekeeping gene; paired Student's t test). (G) Culture of FACS-sorted Lin^−^CD90^−^ALDH^high^ and Lin^−^CD90^−^ALDH^low^ cells showed differences in morphology. Scale bars 100 μm. ^∗^p < 0.05, ^∗∗^p < 0.01, ^∗∗∗∗^p < 0.0001; n.s., not significant.

The mammosphere assay is frequently used to maintain stem cells *in vitro* and is often regarded as a surrogate for CSC content (Stingl et al., 2006). Spheres in the MMTV-PyMT model are composed of different cell types, including CD24^+^CD90^+^ cells (Figure 2A). To better characterize Lin^−^CD24^+^CD90^+^ cells, we sorted them by FACS from tumors and performed qPCRs and cytospins, which showed that in the CD24^+^ fraction CD90 is expressed in a population enriched in mesenchymal-like cells (Figures 2B–2D). Accordingly, Lin^−^CD24^+^CD90^+^ cells FACS sorted from MMTV-PyMT tumors have little sphere-formation ability, while most of the sphere-formation capacity is found in the Lin^−^CD90^−^ALDH^high^ population (Figures 2E and S2). Interestingly, qPCR analyses on FACS-sorted Lin^−^CD90^−^ALDH^high^ and Lin^−^CD90^−^ALDH^low^ cells showed that the latter had a slightly more mesenchymal phenotype than Lin^−^CD90^−^ALDH^high^ cells (Figure 2F). Consistently, when we FACS sorted and grew both populations *in vitro*, Lin^−^CD90^−^ALDH^low^ cells showed a tendency to become more mesenchymal whereas Lin^−^CD90^−^ALDH^high^ cells formed epithelial colonies (Figure 2G). Taken together, these results indicate that in our model, sphere formation is associated with TIP-retaining epithelial progenitors.

### Inhibition of TGFBR1 Produces More Sphere-Forming Cells

**Figure 3:**
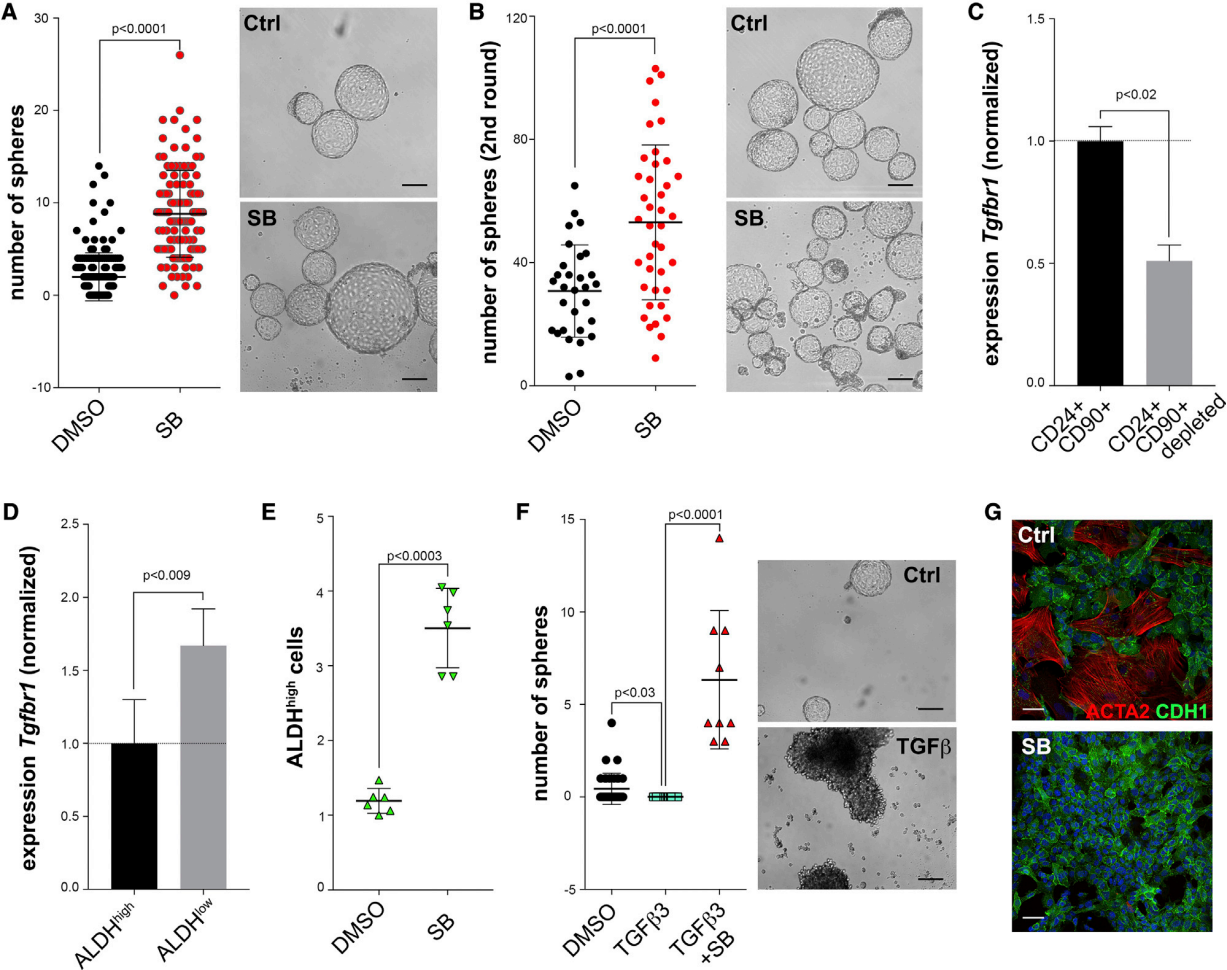
Inhibition of TGFBR1 Leads to an Increase in the Number of TICs (A) Tumor cells were obtained from fresh MMTV-PyMT tumors, grown overnight in collagen-coated plates, and seeded as spheres (10^4^ cells/well) in the presence of 2 μM SB431542 or dimethyl sulfoxide (DMSO). Spheres were counted 10 days later (n = 164 control and n = 122 SB, for six independent tumors; unpaired Student's t test). Scale bars, 100 μm. (B) Primary spheres were trypsinized, counted, and replated in ultralow-attachment plates. Spheres were counted 10 days later (n = 30 not pretreated and n = 40 pretreated, for three independent experiments; unpaired Student's t test). Scale bars, 100 μm. (C and D) qPCR analysis of *Tgfbr1* on FACS-sorted populations of MMTV-PyMT tumors using the CD24CD90 markers (C) or the AldeFluor assay (D) (n = 3 and n = 4 independent tumors, respectively, paired Student's t test). (E) MMTV-PyMT tumors were digested and cells were plated and treated for 48 h with 2 μM SB431542 or DMSO. FACS analyses showed that TGFBR1 inhibition increased the frequency of ALDH^high^ cells (n = 6 independent tumors, paired Student's t test). (F) Tumor cells were obtained from fresh MMTV-PyMT tumors, grown overnight in collagen-coated plates, and seeded as spheres (10^4^ cells/well) in the presence of DMSO, 1 ng/mL TGFβ3, or 1 ng/mL TGFβ3 and 2 μM SB431542. The number of spheres was determined after 10 days (n = 34 control, n = 20 TGFβ3, n = 9 TGFβ3+SB, for two independent tumors; one-way ANOVA and Fisher's LSD). Scale bars, 100 μm. (G) Immunofluorescent staining for E-cadherin (CDH1) and α-smooth muscle actin (ACTA2) in cultured PyMT cells treated with 2 μM SB431542 or DMSO for 5 days. Scale bars, 50 μm.

The acquisition of mesenchymal features through the EMT has been linked to the CSC phenotype (Mani et al., 2008). Since we had observed differences in epithelialization between metastatic CSCs and TICs, we next reasoned that blocking EMT might alter the proportions of CSCs in our system. Therefore, we treated MMTV-PyMT cells with a TGFBR1 inhibitor (SB431542). Surprisingly, treating the cells with the small molecule significantly increased sphere formation (Figure 3A). The same effects were achieved with the structurally different TGFBR1 inhibitor Ly2157299 (Figure S3A). In secondary sphere cultures, the tendency was maintained and the inhibitor still generated more spheres (Figure 3B). We observed similar results in other systems such as 4T1 and MMTV-Wnt1 cells (Figures S3B and S3C). Importantly, we also observed increased sphere formation in two out of three human breast cancer samples treated with SB431542 or Ly2157299 (Figures S3D–S3F). Conversely, adding TGFβ3 reduced sphere formation in all the models we tested (Figure S3). To investigate potential differences in the sensitivity to the inhibitor, we performed qPCR analyses on FACS-sorted MMTV-PyMT tumor cells using either the Lin^−^CD24CD90 or the Lin^−^CD90^−^ALDH strategy. Interestingly, our results show that *Tgfbr1* expression is higher in Lin^−^CD24^+^CD90^+^ and Lin^−^CD90^−^ALDH^low^ cells when compared with Lin^−^CD24^+^CD90^−^ and Lin^−^CD90^−^ALDH^high^ cells, respectively (Figures 3C and 3D), which indicates that these subsets may be more sensitive to TGFBR1 inhibition. Furthermore, upon treating the cells with Noggin or transducing them with a secreted decoy receptor that acts as dominant negative form of the TGFBR2 (Zhao et al., 2002), we showed that the effects of the SB431542 on CSCs are mediated through TGFBR1/ALK5 and not ACVR1B/ALK4 or ACVR1C/ALK7 inhibition (Figures S3G and S3H; data not shown for Nodal). We next analyzed the proportion of ALDH^high^ cells upon treatment and found that TGFBR1 inhibition increased their percentage (Figure 3E), which is consistent with our previous results. In agreement with EMT causing a loss of TICs, treating the cells with TGFβ3 inhibited sphere formation, while this was rescued by addition of SB431542 (Figures 3F and S3I). Consistent with these observations, the inhibitor caused a significant reduction in mesenchymal traits as seen by immunostaining in attached cultures (Figure 3G). Overall, these results indicate that blocking TGFβ signaling through TGFBR1 inhibition is sufficient to trigger the expansion of tumor-initiating progenitors *in vitro*.

### TGFβ Signaling Inhibition Reduces Metastasis but Not Tumor Initiation

**Figure 4:**
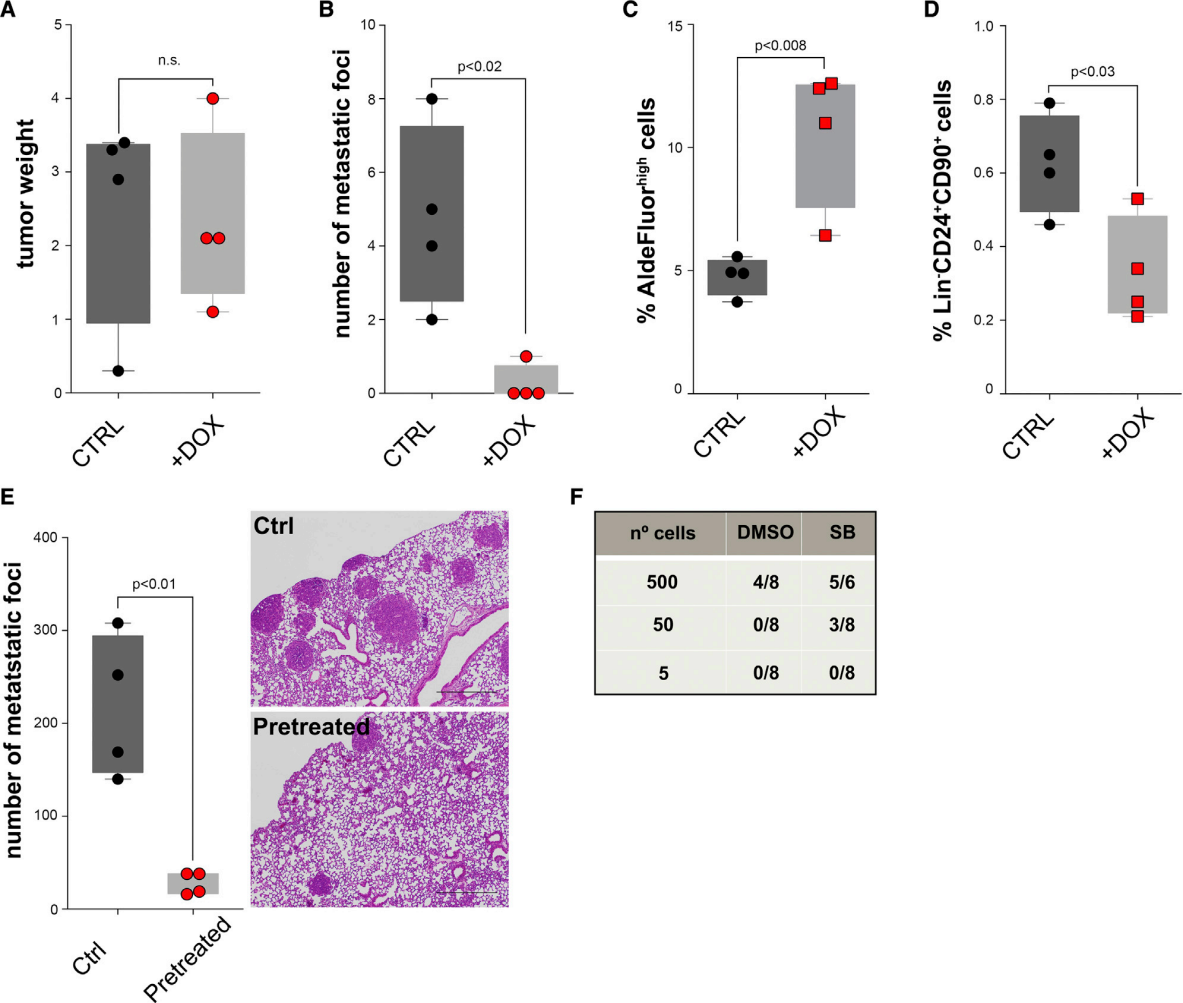
Inhibition of TGFBR1 Decreases Metastasis but Increases Tumor-Initiation Potential (A and B) MMTV-PyMT cells infected with a shTgfbr1 were grafted orthotopically into FVB/N mice, and one group was given doxycycline (DOX; 1 mg/mL) in the drinking water for the duration of the experiment. Both groups formed equally sized tumors (A), but downregulation of *Tgfbr1* led to a significant decrease in lung metastases (B) (n = 4, unpaired Student's t test). (C and D) Tumors in which *Tgfbr1* was downregulated showed increased frequencies of Lin^−^ALDH^high^ cells (C; n = 4, unpaired Student's t test) but a reduced number of Lin^−^CD24^+^CD90^+^ cells (D; n = 4, unpaired Student's t test). (E and F) Cells from fresh MMTV-PyMT tumors were plated and pretreated with either 2 μM SB431542 or DMSO for 5 days and thereafter injected via tail vein or othotopically in limiting dilution assays in FVB/N mice. Pretreating the cells with SB431542 resulted in decreased metastatic colonization potential upon tail vein injection (E; n = 4, unpaired Student's t test; scale bars, 500 μm), while orthotopic injection in limiting dilution assays revealed that pretreated cells exhibit higher TIP (F).

We next produced three inducible short hairpin RNAs (shRNAs) for *Tgfbr1* (1,535, 825, and 777, Figure S4A) to validate our results *in vivo*. We infected PyMT cells with shTgfbr1 lentiviruses and injected them orthotopically in FVB/N mice. While tumors did not differ in size, we observed a significant reduction in metastasis in those animals with tumors in which we downregulated *Tgfbr1* by doxycycline-induced shRNA expression (Figures 4A and 4B). Similar results were obtained using 4T1 cells in BALB/c mice (Figures S4B and S4C). Downregulating *Tgfbr1* in PyMT cells was consistently sufficient to increase the proportion of Lin^−^ALDH^high^ cells and reduce that of Lin^−^CD24^+^CD90^+^ *in vivo* (Figures 4C and 4D, respectively). Finally, we treated PyMT cells with SB431542 and injected them either via tail vein or orthotopically in limiting dilution assays. Cells that were pretreated showed reduced metastatic ability in lung metastasis assays (Figure 4E), but had higher TIP (Figure 4F, p < 0.03). Of note, SB431542 does not affect cell viability *in vitro* (Figure S4D). Taken together, these results indicate that TGFβ signaling regulates metastatic and tumor-initiating CSC.

## Discussion

CSCs are defined as a subset of cells in a tumor, which possess stem cell properties and sustain tumor growth. These properties are inherent to some particular cells within the tumor, but can also be acquired, which reflects a certain degree of cellular plasticity (Batlle and Clevers, 2017). In agreement with this rather broad definition, different subsets of CSCs have been identified in different tumors including breast (Yeo et al., 2016). Furthermore, plasticity in the stem cell niche is known to be an important factor regulating transitions to different stem-like states in CSCs (Brooks et al., 2015). The CSC field suffers from a major drawback, namely the lack of universal markers: they can vary from mouse to human and between different models of the same type of cancer. Ginestier et al. (2007) showed that in human breast cancer, the overlap between ALDH^high^ and Lin^−^CD44^+^CD24^−^ cells is small (1.16%), and that most of the TIP is retained by the ALDH^high^non(CD24^−^CD44^+^) and ALDH^high^CD24^−^CD44^+^ fractions, while ALDH^low^CD24^−^CD44^+^ have little TIP. Nevertheless, this isolation strategy (CD24CD44) cannot be formally compared with ours (CD24CD90), because it is not known whether CD44^+^CD24^−^ are bona fide metastatic stem cells. Likewise, whether the presence/abundance of CD24^−^CD44^+^ predicts poor prognosis or is associated with distant metastasis is a matter of debate (Abraham et al., 2005, Mylona et al., 2008, Nogi et al., 2011, Wei et al., 2012). We here used the MMTV-PyMT model to show that two populations of tumor cells with distinct tumor-initiating abilities coexist within mammary tumors. The first, which we described as retaining most metastatic potential in the tail vein injection assay, has mesenchymal features and is defined as Lin^−^CD24^+^CD90^+^ (Malanchi et al., 2012). In addition, we now found the lineage-restricted Lin^−^CD90^−^ALDH^high^ epithelial-like population to be highly enriched in cells with TIP but with poorer metastasis-initiating capacity. As expected, grafting the same tumor cells in NSG or FVB mice produced significant differences in the estimated stem cell frequency. When grafted in NSG mice, Lin^−^CD90^−^ALDH^high^ cells had an estimated 14.3% of TICs while in FVB, the figure dropped to 0.8%. These results suggest that in the MMTV-PyMT model, approximately 94% of the tumor cells showing tumor-initiation capacity when grafted in NSG mice are killed by T or NK cells when grafted into immunocompetent mice, and therefore cannot be considered bona fide CSCs (Bruttel and Wischhusen, 2014). This issue has been observed by a number of groups and underlines the importance of using syngeneic models in immunocompetent mice (Quintana et al., 2008).

To understand how these subsets of CSCs are controlled, we modulated TGFβ signaling and found that it shifts the proportions of these two CSC populations. Not surprisingly, blocking TGFβ signaling inhibits the metastatic population and interferes with the metastatic cascade. However, it also triggers the expansion of ALDH^high^ cells, which have a high tumor-initiation capacity. Our results are in contrast to those previously published showing that in pancreatic cancer TGFβ inhibition decreases CSC numbers, including sphere-forming cells (Donahue and Dawson, 2011). However, it is well known that in breast cancer TGFβ plays pleiotropic roles that are context dependent and, therefore, this may be one of the reasons explaining the differences observed (David and Massague, 2018, Fang et al., 2013). Bhola et al. (2013) showed that in combination with paclitaxel, TGFβ inhibition decreased the frequency of triple-negative breast cancer TICs. Interestingly, their data indicate that treating SUM159 cells with the small molecule Ly2157299 increased ALDH^+^ cells and sphere formation, both surrogates for TIP, which is in agreement with our results. In a recent work, Beerling et al. (2016) suggested that cellular plasticity uncouples the effects of EMT on CSCs. Our results show that, in our model, secondary and primary TIP are features not necessarily shared by the same cell type that can be regulated by TGFBR1 inhibition. These data indicate that TGFBR1 inhibitors might exert different effects that are cell and context dependent. These results may have important implications for treatment, since TGFβ inhibitors are currently under clinical trials. In particular, it might be relevant for those patients with circulating tumor cells (CTCs). The presence of CTCs in patients with breast cancer is associated with bad prognosis (Cristofanilli et al., 2005). It was recently shown that metastases can be polyclonal, i.e., arising from multiple clones that seed the secondary site (Cheung et al., 2016). This is in line with previous results suggesting that CTC clusters are the precursors of polyclonal metastases (Aceto et al., 2014). Our data suggest the possibility that treatment with TGFβ inhibitors might promote the expansion of circulating tumor stem-like cell clusters, and therefore advise caution when using them to treat patients with breast cancer.

## Experimental Procedures

Detailed methods for FACS, qPCR, western blot analysis, and cell culture are provided in Supplemental Information.

### Mouse Work

MMTV-PyMT (FVB/N) mice were bred and housed in ventilated cages in the OHB mouse husbandry of the University of Fribourg. For PyMT tumor cell transplantation to the fourth mammary fat pad or tail vein injection experiments, we used NSG and immunocompetent FVB/N mice. The experiments involving 4T1 cell injections were done in immunocompetent BALB/c mice. For limiting dilution experiments, cells were injected in Matrigel/PBS (1:3). All the experimental procedures involving mice were carried out in accordance with the Swiss Animal Welfare Regulations and were previously approved by the Cantonal Veterinary Service of the Canton Fribourg (2017_26_FR).

### Statistics

The results were analyzed using GraphPad Prism 7 software. Means were compared with either paired or unpaired Student's t test. In case groups would not pass a normality test (assessed using D'Agostino-Pearson's omnibus normality test), samples were analyzed with the Mann-Whitney non-parametric test. When comparing more than two variables, we performed one-way ANOVA. To isolate differences between groups, we performed Fisher's least significant difference (LSD) test. p values are indicated for each experiment. Limiting dilution assay data were analyzed using ELDA (extreme limiting dilution assay) (Hu and Smyth, 2009). Experiments were done at least in triplicate. Error bars indicate standard deviation. Significant differences between experimental groups are indicated with asterisks in the figures as follows: ^∗^p < 0.05, ^∗∗^p < 0.01, ^∗∗∗^p < 0.001, and ^∗∗∗∗^p < 0.0001.

## Author Contributions

Conceptualization, A.S.-M.; Methodology, A.S.-M. and F.F.; Investigation, F.F., A.S.-M., and M.B.; Formal Analysis, F.F. and A.S.-M.; Resources, A.S.-M. and C.R.; Writing – Original Draft, A.S.-M. and F.F.; Writing – Review & Editing, A.S.-M. and F.F.; Funding Acquisition, A.S.-M.; Supervision, A.S.-M. All authors read and approved the final manuscript.

## Supplemental Information

Document S1. Supplemental Experimental Procedures, Figures S1–S4, and Table S1

Document S2. Article plus Supplemental Information
