# Supplementary material for: Breast Cancer Stem Cells with Tumor- versus Metastasis-Initiating Capacities Are Modulated by TGFBR1 Inhibition
Source: Stem Cell Reports. 2019 Jun 27;13(1):1–9. doi: 10.1016/j.stemcr.2019.05.026 (PMC6626885; doi:10.1016/j.stemcr.2019.05.026)
Supplement: Document S1. Supplemental Experimental Procedures, Figures S1–S4, and Table S1 [file mmc1.pdf]

Stem Cell Reports, Volume *13*

Supplemental Information

Breast Cancer Stem Cells with Tumor- versus Metastasis-Initiating Capacities Are Modulated by TGFBR1 Inhibition

Flavia Fico, Mélanie Bousquenaud, Curzio Rüegg, and Albert Santamaria-Martínez

Inventory of Supplemental Information

Supplemental Figure 1. Metastatic stem cells vs tumor-initiating cells. Related to Figure 1.

Supplemental Figure 2. Characterization of CSC. Related to Figure 2.

Supplemental Figure 3. Inhibition of TGFBR1 leads to an increase in the number of TIC. Related to Figure 3.

Supplemental Figure 4. Inhibition of TGFBR1 decreases metastasis but increases TIP. Related to Figure 4.

Supplemental Experimental Procedures

- Antibodies and reagents
- Mouse work extended
- Orthotopic transplants
- Tail vein injections
- Cell culture
- Tumor sphere assays
- Lentiviral production
- FACS analysis
- Immunostaining
- Western blot
- Real-time PCR
- MTT assay

Supplemental File 5. Table 1: Primer sets used in this study.

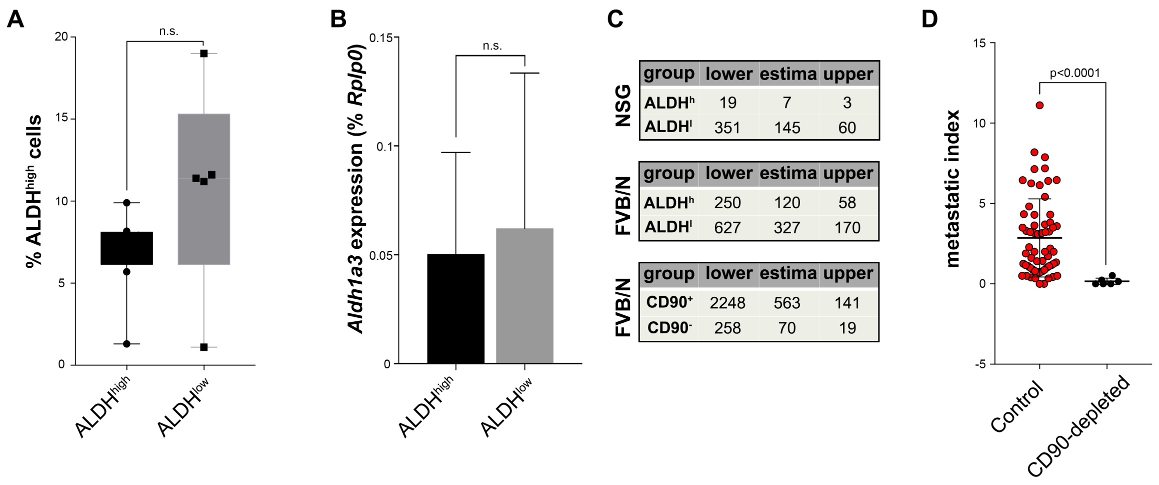

Supplemental Figure 1. Metastatic stem cells vs tumor-initiating cells. Related to Figure 1. (A) Tumors generated using FACS-sorted ALDH^{high} and ALDH^{low} MMTV-PyMT cells were digested and re-analysed again using the AldeFluor assay (n=5 independent tumors, Mann-Whitney test). (B) Tumors generated using FACS-sorted ALDH^{high} and ALDH^{low} were pulverised in liquid nitrogen, RNA was extracted and qPCRs were performed to evaluate the expression of *Aldh1a3* transcripts (n=5 independent tumors, unpaired Student's t test). (C) Cancer stem cell frequency with confidence intervals of ALDH^{high} and ALDH^{low} cells or CD24^{+}CD90^{+}/CD24^{+}CD90^{-} shown in Figure 1B, 1D and 1E. (D) Metastatic index of CD90-depleted tumors vs CD90^{+} cells containing tumors was calculated as the tumor weight (g) divided by the number of metastatic foci in the lungs (n=62 control and n=6 CD90-depleted independent tumors, Mann-Whitney test).

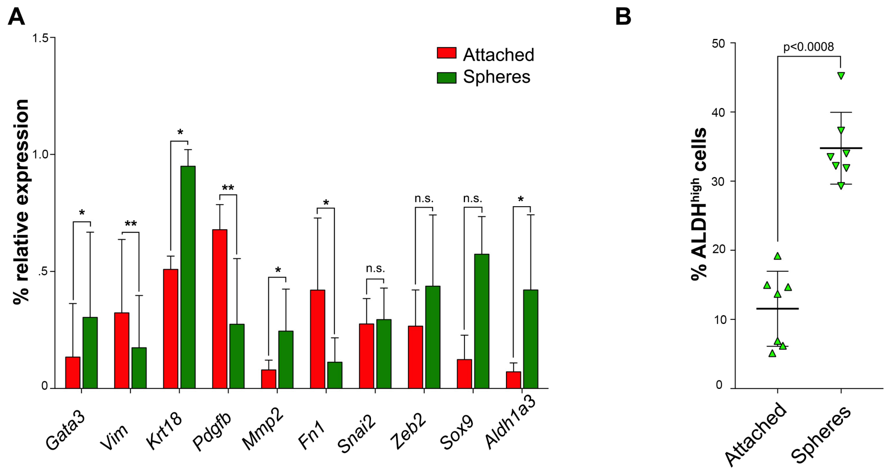

Supplemental Figure 2. Characterization of CSC. Related to Figure 2. (A) qPCR comparing attached vs sphere cultures of MMTV-PyMT cells derived from the same tumors (n=8 independent tumors, paired Student's t test). (B) MMTV-PyMT tumors were digested and cells were plated either in 2D or 3D cultures. FACS analyses show that tumors plated as spheres have a higher frequency of $ALDH^{high}$ cells (n=7 independent tumors, paired Student's t test).

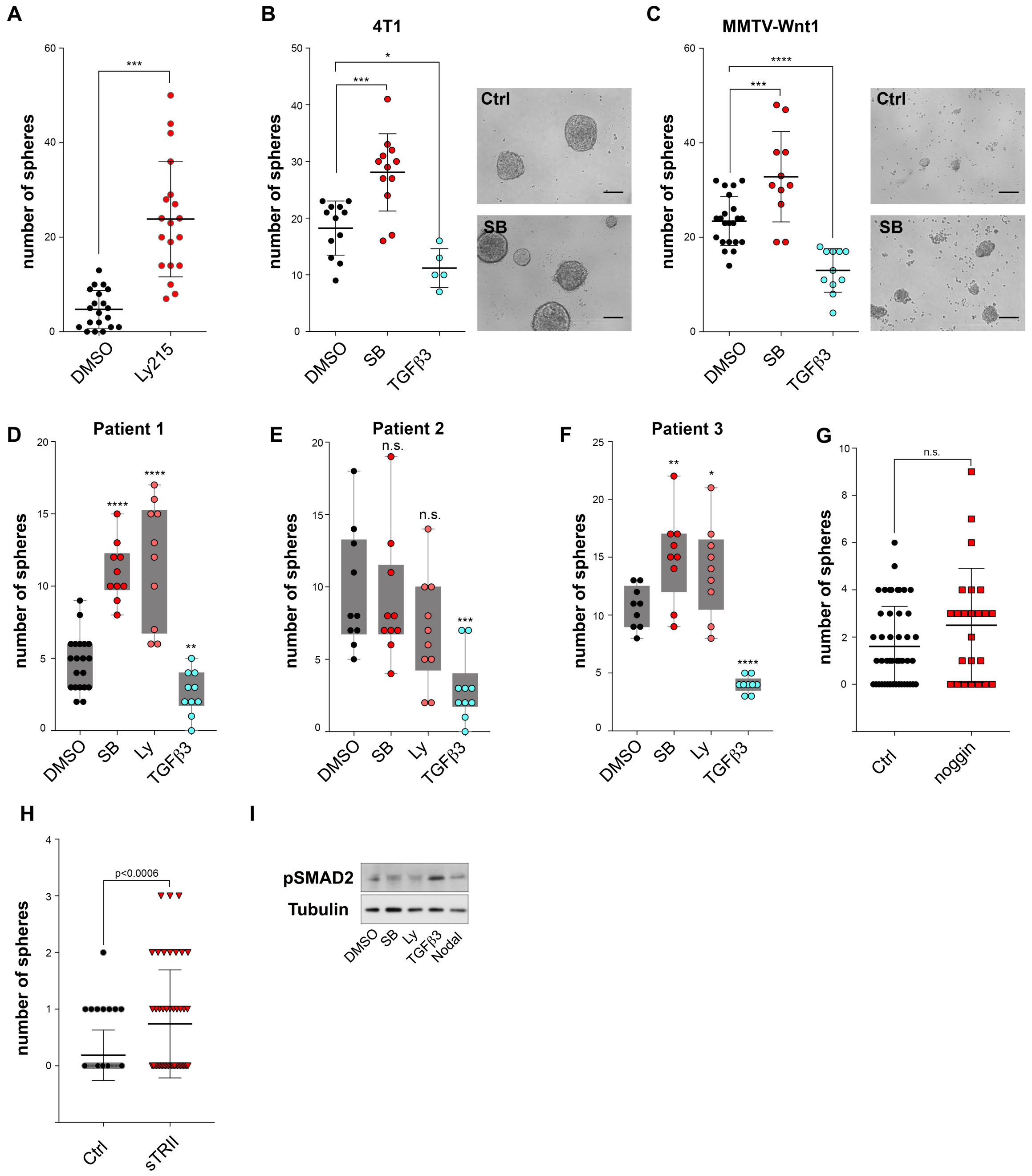

Supplemental Figure 3. Inhibition of TGFBR1 leads to an increase in the number of TIC. Related to Figure 3. (A) Tumor cells were obtained from fresh MMTV-PyMT tumors, grown overnight in collagen-coated plates and then seeded as spheres (10^4 cells/well) in presence of either 2 µM Ly2157299 or DMSO. Spheres were counted 10 days later (n=20 DMSO, n=19 Ly, for 3 independent tumors, unpaired Student's t test). (B, C) 4T1 (n= 12 DMSO, n=12 SB, n=5 TGFβ3) and MMTV-Wnt1 cells (n=22 DMSO, n=11 SB, n=11 TGFβ3) were plated as spheres (10^4 or $5x10^3$ cells/well and 10^4 cells/well, respectively) and treated with 2 µM SB431542, 1 ng/ml TGFβ3 or DMSO. Spheres were counted 7-10 days later (One-way ANOVA and Fisher's LSD, scale bar 100 µm). (D-F) Human breast cancer tissue was digested with collagenase and seeded directly in ultralow attachment plates with media containing DMSO, 2 µM SB431542, 2 µM Ly2157299 or 1 ng/ml TGFβ3. Spheres were counted one week later (One-way ANOVA and Fisher's LSD). (G) Tumor cells were obtained from fresh MMTV-PyMT tumors, grown overnight in collagen-coated plates and then seeded as spheres (10^4 cells/well) in presence or absence of 500 ng of noggin. Spheres were counted 10 days later (n=48 DMSO, n=24 noggin, for 4 independent tumors, unpaired Student's t test). (H) Tumor cells were obtained from fresh MMTV-PyMT tumors, grown overnight in collagen-coated plates, trypsinized and infected with lentiviruses containing either the mock or a TGFBR2 decoy receptor (sTRII), selected and seeded in ULA plates. Spheres were counted 10 days later (n=48 ctrl, n=46 sTRII, for 2 independent tumors, unpaired Student's t test). (I) Spheres were collected by centrifugation and protein extracts were subjected to Western blot analysis of pSMAD2 to test the TGFβ pathway activation.

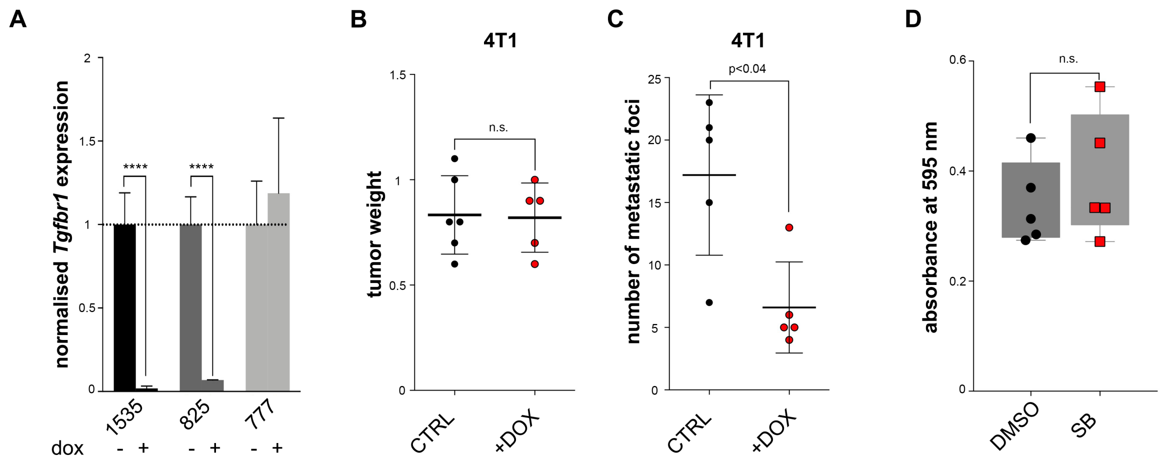

Supplemental Figure 4. Inhibition of TGFBR1 decreases metastasis but increases tumor initiation potential. Related to Figure 4. (A) Fresh MMTV-PyMT tumor-derived cells were infected in suspension with lentiviruses containing the *Tgfbr1* short hairpins. The expression was induced treating the cells with with 1µg/ml doxycycline for 48h. The graphs show the normalized mRNA expression levels for *Tgfbr1* in activated vs non-activated cells. (B, C) 4T1 cells infected with a shTgfbr1 were grafted orthotopically into BALB/c mice and one group was given doxycycline (1 mg/ml) in the drinking water for the duration of the experiment. Both groups formed equally sized tumors, but downregulation of *Tgfbr1* led to a significant decrease in lung metastases (n=5, unpaired Student's t test). (D) MMTV-PyMT cells were assayed for MTT upon treatment with either 2 µM SB431542 or DMSO (n=5 independent tumors, paired Student's t test).

Supplemental Experimental Procedures

Antibodies and reagents

CD90.1 (HIS51), CD90.2 (53-2.1), CD24 (M1/69), eBioscience; Ter119, CD31 (MEC13.3), CD45 (30-F11), Biolegend; Cytokeratin 14 (Covance); pSMAD2, E-Cadherin (Cell Signaling); α-SMA, β-Tubulin (Sigma); Vimentin (Lifespan Biosciences); SB431542 (Stem Cell Technologies); Ly2157299 (Selleckchem), TGFβ (Promega). Both small molecules were dissolved using DMSO and stocked at 10 mM. The plasmids containing the short hairpins for Tgfbr1 were produced by classical molecular cloning and subcloned into pRRL vectors containing a doxycycline inducible *tet* system and a blasticidin resistance gene for mammalian selection. The sequences of the hairpins are (5'-3'): Alk5_1535: TTAATTCGCAAAGCTGTCAGCC; Alk5_777: AGAAGAGAATATCTTCACAGCA; Alk5_825: TACAGTCTGATAAATCTCTGCC.

Mouse work extended

Orthotopic transplants

To generate orthotopic transplants of MMTV-PyMT tumor cells, FVB/N mice were injected with 10^6 PyMT cells resuspended in 50 µl of PBS in the 4th mammary fat pad using an insulin syringe (26G). 4T1 tumors were generated by injecting $2.5x10^5$ cells in the 4th mammary fat pad of BALB/c mice using an insulin syringe (26G). Metastatic foci in the lungs were counted using a Leica M125 stereomicroscope.

Tail vein injections

Mice were warmed by placing the cage under an IR light bulb. One mouse at a time was placed in a tube rodent holder for tail vein injection, with the tail outside of the tube. The tail was cleaned with 70% ethanol. The IR light bulb was placed above the tail to cause the veins to dilate. MMTV-PyMT tumor cells ($5x10^5$ per mouse) were resuspended in PBS and injected very slowly in a 100 µl volume into one of the two tail veins using an insulin syringe (26G needle). The spot of injection was then compressed with a tissue to make sure the tail was not bleeding. Mice were returned to the cage and kept for observation for 15 min. Metastatic foci in the lungs were counted after 3-5 weeks using a Leica M125 stereomicroscope.

Cell culture

Mouse tumor tissue was dissociated mechanically, followed by an incubation with 1:66 Liberase TH (Roche) and DNAse (10 mg/ml) at 37ºC for 1 hour. Cells were then washed twice in 2 mM EDTA in PBS and once in PBS and then plated in collagen-coated plates (HBSS, BSA 100 mg/ml, HEPES 1M pH 6.5 and bovine collagen biomatrix by Cell Systems). Cells were grown in DMEM:F12 (Gibco) supplemented with 2% FBS, 1% penicillin/streptomycin 20 ng/ml EGF (Invitrogen) and 10 µg/ml insulin (Invitrogen) and let attach overnight. 4T1 cells were obtained from the ATCC and grown as recommended. Human breast tumor tissue was obtained under informed consent from patients undergoing breast surgery (tumorectomy or mastectomy) for an untreated breast cancer. Tissues were collected in HBSS+2% FBS+1% penicillin/streptomycin. They were then digested mechanically and enzymatically with a 0.3% collagenase IV (Sigma) containing solution for 1 hour at 37ºC.

Tumor sphere assays

Sphere cultures were established from fresh total tumor cell preparations. After dissociation, tumor cells were plated on collagen-coated plates overnight, trypsinized next day and plated in 150 µl of sphere media (DMEM/F12 with B27, 20 ng/ml EGF, 20 ng/ml FGF, 4 µg/ml heparin, 1% penicillin/streptomycin) with or without the small molecules at the indicated concentrations into 96-well low attachment plates (Cornig) at $1x10^4$ cells per well and at least 5 wells per tumor. MMTV-PyMT spheres were counted after one week (7-10 days). Secondary mammosphere culture was performed by collecting the spheres through gentle centrifugation (800 rpm) followed by enzymatic (10 min in 0.05% trypsin-EDTA at 37°C) mechanical dissociation using a pipette. Mammospheres were stained with the indicated antibodies and images were taken with an inverted confocal microscope.

Lentiviral production

Lentiviral particles were produced in HEK293T cells by calcium phosphate precipitation. Briefly, two hours prior to transfection, $11x10^6$ HEK293T cells/15 cm dish were incubated with DMEM+10%FBS supplemented with 25 µM chloroquine (Sigma). Lentiviruses were produced by cotransfection of HEK293T cells with the vectors of interest together with the pCMV-dR8.74 and the pMD2G (VSVG). Next day, media was removed and replaced with fresh media containing 3 mM caffeine (Sigma). On the third day, the supernatant containing the viral particles was collected, ultracentrifuged for 2.5 hours at 20000 rpm to concentrate the lentiviruses and used to infect cells or aliquoted and stored at -80ºC.

FACS Analysis

For FACSorting experiments, tumor cells derived from MMTV-PyMT mice were obtained by enzymatic disaggregation as described above. Cells were then washed twice with PBS, filtered (0.70 μM filters), stained with the appropriate antibodies for 30 minutes at 4ºC and sorted using either a FACSAria, a FACSAria III (BD Biosciences) or a MoFlo Astrios (Beckman Coulter). For FACS analysis, tumor cells were trypsinized, washed and stained with the appropriate antibodies for 30 minutes at 4ºC. DAPI or 7-AAD were used to discard dead cells. ALDH activity was tested using the AldeFluor™ assay kit (Stem Cell Technologies) as per the manufacturer's protocol. Briefly, cells were incubated with either the AldeFluor™ reagent alone or the AldeFluor™ reagent and the inhibitor diethylaminobenzaldehyde (DEAB) for 30 minutes at 37ºC. Cells were then centrifuged, washed and immunophenotyped when required. Fluorescence was analysed using either a Cyan ADP (Dako) or a MACSQuant (Miltenyi) instrument. Data was processed and analysed using FlowJo.

Immunostaining

MMTV-PyMT cells were grown on glass cover-slips, treated with vehicle (DMSO) or 2 μM SB431542 for 5 days and then fixed with 4% PFA for 15 minutes and subsequently washed with PBS. The unspecific binding of the antibodies was blocked using a solution containing 10% serum for 1 hour at room temperature. Samples were then incubated with primary antibodies for 1 hour at room temperature, washed 3 times with 1x PBS and incubated with a secondary antibody conjugated to AlexaFluor-488 for 1 hour at room temperature in a humid-chamber protected from light. Cell nuclei were stained with DAPI. Fluorescent images were taken with a LSM700 inverted confocal microscope (Zeiss), setting the pinhole at 1 AU.

Western blot

Protein was extracted with complete RIPA buffer (20 mM Tris-HCl (pH 7.5), 150 mM NaCl, 1 mM Na2EDTA, 1 mM EGTA, 1% NP-40, 1% sodium deoxycholate, 2.5 mM sodium pyrophosphate, 1 mM β-glycerophosphate, 1 mM Na3VO4, 1 μg/ml leupeptin; Cell Signaling), separated by electrophoresis, transferred to PVDF membranes (Millipore), blocked with 5% BSA (Carl Roth) in 0.1% Tween 20 containing Tris-buffered saline (TBST) and incubated overnight with primary antibodies. Immunoreactive bands were visualized using HRP-conjugated secondary antibodies (Cell Signaling and Dako).

Real-time PCR

RNA was prepared using the mini or micro RNA kit (Qiagen) as per the manufacturer's instructions. cDNAs were generated using oligo-T priming and the M-MLV Reverse Transcriptase RNase H (-) Point Mutant (Promega). qPCR was performed in a StepOnePlus thermocycler (Applied Biosystems) using the SYBR green PCR Master Mix (Kapa) and following the manufacturer's instructions. A list of primers used is shown in Supplemental File 5: Table 1.

MTT assay

MTT [3-(4,5-Dimethylthiazol-2-yl)-2,5-Diphenyltetrazolium bromide] solution was prepared at 5 mg/ml in PBS. MMTV-PyMT cells were plated in 96 well plates in DMEM/F12 supplemented with 2% FBS, 20 ng/ml EGF, 10 μg/ml insulin and 1/10 vol of MTT solution was added to the medium. Cells were incubated for 4 hours at 37ºC, then media was removed and replaced with 200 μl of DMSO. The dye was completely dissolved by pipetting and the solution was incubated for 5 minutes at RT. Optical density was measured in a plate reader at 570 nm.

Supplemental File 5. Table 1: Primer Sets Used in This Study.

ID	Forward 5'-3'	Reverse 5'-3'
Rplp0	GATTCGGGATATGCTGTTGG	GTTCTGAGCTGGCACAGTGA
Krt18	CGAGGCACTCAAGGAAGAAC	AATCTGGGCTTCCAGACCTT
Tgfbr1	AAATTGCTCGACGCTGTTCT	CAACCGATGGATCAGAAGGT
Aldh1a3	GAGCGATCCTGGCTACTCTG	GACGAAAAAGGCATGAAGGA
Sox9	CAGCAAGACTCTGGGCAAG	TCCACGAAGGGTCTCTTCTC
Gata3	GCTACGGTGCAGAGGTATCC	AGAGATCCGTGCAGCAGAG
Mmp2	ACACTGGGACCTGTCACTCC	TGTCACTGTCCGCCAAATAA
Mmp9	CCAGATGATGGGAGAGAAGC	TTGAGGCCTTTGAAGGTTTG
Snai2	GAACCCACACATTGCCTTGT	GCAGAAGCGACATTCTGGAG
Zeb2	CTATTCCCCTGCATCAGCAT	GGCTTGTCAGTCCTTTCTCG
Vim	GCGAGGAGAGCAGGATTTCTC	GGGTGTCAACCAGAGGAAGT
Fn1	TGCACGTGTGTGGGGAACGG	CCCGGCCCTGACCAAAGCAG
Pdgfb	TGGTATCACTCCTGGAAGCC	AACAGAAGACAGCGAGGTGG
