# Supplementary material for: Breast Cancer Stem Cells with Tumor- versus Metastasis-Initiating Capacities Are Modulated by TGFBR1 Inhibition
Source: Stem Cell Reports. 2019 Jun 27;13(1):1–9. doi: 10.1016/j.stemcr.2019.05.026 (PMC6626885; doi:10.1016/j.stemcr.2019.05.026)
Supplement: Document S2. Article plus Supplemental Information [file mmc2.pdf]

Stem Cell Reports

Report

ISSCR

OPEN ACCESS

Breast Cancer Stem Cells with Tumor- versus Metastasis-Initiating Capacities Are Modulated by TGFBR1 Inhibition

Flavia Fico,[1] Mélanie Bousquenaud,[2] Curzio Rüegg,[2] and Albert Santamaria-Martínez[1,*]
[1]Tumor Ecology Lab, Department of Oncology, Microbiology and Immunology, Faculty of Science and Medicine, University of Fribourg, Fribourg, Switzerland
[2]Experimental and Translational Oncology Lab, Department of Oncology, Microbiology and Immunology, Faculty of Science and Medicine, University of Fribourg, Fribourg, Switzerland
*Correspondence: albert.santamaria@unifr.ch
https://doi.org/10.1016/j.stemcr.2019.05.026

SUMMARY

Cancer stem cells (CSCs) are defined by their ability to regenerate a tumor upon transplantation. However, it is not yet clear whether tumors contain a single CSC population or different subsets of cells with mixed capacities for initiating primary and secondary tumors. Using two different identification strategies, we studied the overlap between metastatic stem cells and tumor-initiating cells (TICs) in the MMTV-PyMT model. Our results show that in the MMTV-PyMT model, Lin$^-$CD90$^-$ALDH^{high} cells retained a high tumor-initiating potential (TIP) in orthotopic transplants, in contrast to Lin$^-$CD24$^+$CD90$^+$, which retained higher metastatic capacity. Interestingly, suppression of TGFβ signaling increased TIC numbers. We here describe the existence of distinct populations of CSCs with differing capacities to initiate tumors in the primary or the secondary site. Inhibiting TGFβ signaling shifts the balance toward the former, which may have unanticipated implications for the therapeutic use of TGFβ/TGFBR1 inhibitors.

INTRODUCTION

The cancer stem cell (CSC) hypothesis proposes that, similarly to what happens in normal tissues, heterogeneity within tumors is the consequence of their hierarchical organization, i.e., that many tumors are organized as a pyramid with CSCs at its apex. In many tumor types such as breast cancer, this subset of cells is known to sustain tumor growth but also, as we and others have shown, to lead metastatic colonization (Malanchi et al., 2012). The latter is particularly important since over 90% of cancer-related deaths are due to metastatic disease. Experimentally, CSCs are defined as tumor-derived cells that have the exclusive ability to regenerate a tumor upon transplantation—with all its full complexity and heterogeneity. To assess this capacity, typically CSCs are purified from tumors and tested for their tumor-initiating ability in limiting dilution assays. However, the lack of universal CSC markers poses a serious problem in understanding how homogeneous the CSC pool is.

A few years ago, Weinberg's lab proposed that CSCs can be generated from the epithelial-to-mesenchymal transition (EMT) (Mani et al., 2008), a process that confers motility and invasiveness to cancer cells and therefore is advantageous to metastasis. However, most secondary tumors derived from carcinomas show an epithelial morphology resembling that of the primary tumor. This suggests that metastatic cells may need to undergo a reverse process, the mesenchymal-to-epithelial transition, once they have colonized a secondary organ (Celia-Terrassa et al., 2012; Ocana et al., 2012; Tsai et al., 2012). Interestingly, the use of different isolation strategies in the same model allowed some researchers to distinguish between two subsets of CSCs according to their mesenchymal features: EMT-CSCs and MET-CSCs (Liu et al., 2014). Nevertheless, it is not clear whether CSC transition from one state to the other or two different CSC subpopulations exist in a tumor at a given time. Therefore, understanding whether tumor-initiating cells (TICs) are metastatic is essential in the design of rational targeted therapies and more accurate CSC isolation protocols. Since both tumor-initiating and metastatic CSCs need a different set of features to initiate tumors, we aimed at studying whether they are the same cell population. Here, we use the MMTV-PyMT model to identify two subgroups of CSCs and show that transforming growth factor β (TGFβ) receptor 1 (TGFBR1)/ALK5 inhibition prevents metastasis but not tumor initiation.

RESULTS

CD90$^-$ALDH^{high} Cells Are Lineage-Committed CSCs

The ability of cancer cells to form metastasis in the lungs is typically tested in intravenous injections in the tail vein. Using the MMTV-PyMT model, we have previously shown that most of this capacity is retained by Lin$^-$CD24$^+$CD90$^+$ cells (Malanchi et al., 2012). However, what is not clear is the extent of the overlap, if any, between metastatic stem cells and primary TICs. The AldeFluor assay, which determines aldehyde dehydrogenase (ALDH) activity, is frequently used to identify cells that possess

This is an open access article under the CC BY license (http://creativecommons.org/licenses/by/4.0/).

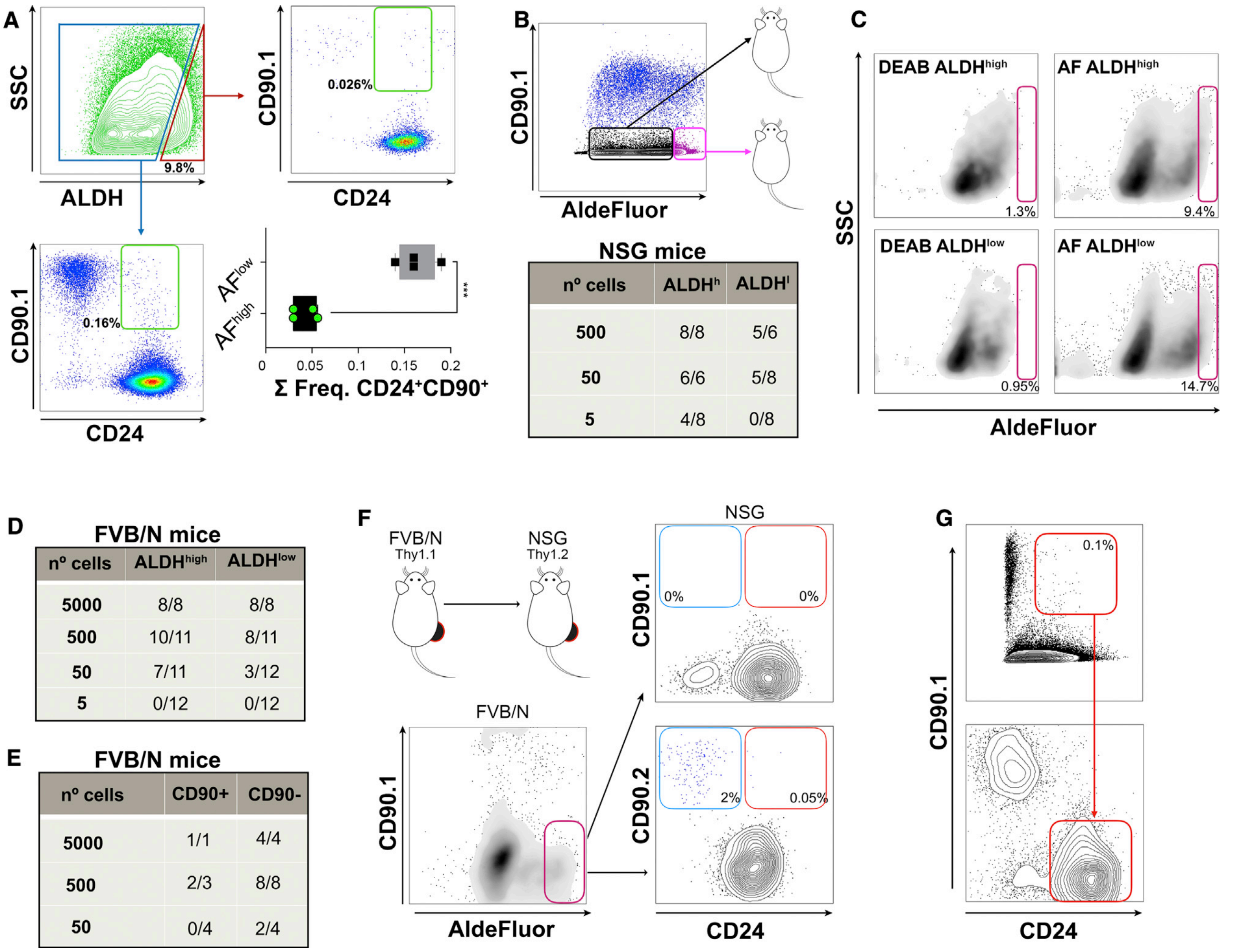

Figure 1. Metastatic Stem Cells versus Tumor-Initiating Cells

(A) FACS analyses of MMTV-PyMT fresh tumors. Lin^{-}ALDH^{high} and Lin^{-}ALDH^{low} DAPI-negative singlets were gated and analyzed for the expression of CD24 and CD90 (absolute frequency, n = 4 independent tumors, paired Student's t test).

(B) MMTV-PyMT cells from fresh tumors were FACS sorted using the AldeFluor assay, then counted and injected orthotopically in limiting dilution assays in NSG mice. The presence or absence of tumors was evaluated for a maximum of 3 months after injection. Data were analyzed using ELDA Extreme.

(C) Cytograms showing the AldeFluor assay profiles of tumors derived from Lin^{-}CD90^{-}ALDH^{high} and Lin^{-}CD90^{-}ALDH^{low} cells.

(D) MMTV-PyMT cells from fresh tumors were FACS sorted using the AldeFluor assay, then counted and injected orthotopically in limiting dilution assays in FVB/N mice. The presence or absence of tumors was evaluated for a maximum of 3 months after injection. Data were analyzed using ELDA Extreme.

(E) MMTV-PyMT cells from fresh tumors were FACS sorted for CD24CD90, then counted and injected orthotopically in limiting dilution assays in FVB/N mice. The presence or absence of tumors was evaluated for a maximum of 3 months after injection. Data were analyzed using ELDA Extreme.

(F and G) Lin^{-}CD90^{-}ALDH^{high} cells from MMTV-PyMT spontaneous tumors (FVB/N, Thy1.1) were FACS sorted and transplanted into NSG (Thy1.2) mice to determine lineage restrictions (F). Note that host-derived cancer-associated fibroblasts are CD90.2. (G) FACS-sorted Lin^{-}CD24^{+}CD90^{+} cells from MMTV-PyMT spontaneous tumors (upper cytogram) can give rise to tumors with Lin^{-}CD24^{+}CD90^{-} cells when transplanted syngeneically (lower cytogram).

higher tumor-initiation capacity (Wan et al., 2014). We therefore performed fluorescence-activated cell sorting (FACS) analyses on PyMT cells, which revealed that there is no significant overlap between Lin^{-}CD24^{+}CD90^{+} cells and Lin^{-}ALDH^{high} cells, suggesting that both strategies identify different populations (Figure 1A). To uncouple the effects on primary tumor- and metastasis-initiation capacity, we next performed orthotopic transplantation of

CD90-depleted cancer cells in limiting dilution assays. Our results show that Lin$^-$CD90$^-$ALDH^{high} cells possessed 20.7-fold higher tumor-initiating potential (TIP) than Lin$^-$CD90$^-$ALDH^{low} ($p < 0.0001$), with an estimated stem cell frequency of 1 in 7 cells when injected in NSG mice (Figure 1B). This indicates that Lin$^-$CD90$^-$ALDH^{high} is a population enriched in bona fide CSCs with TIP. It is worth noting that in those Lin$^-$CD90$^-$ALDH^{low} tumors that are able to grow, ALDH activity is recovered (Figures 1C, S1A, and S1B), which suggests that a subset of Lin$^-$ALDH^{low} cells can replenish the Lin$^-$ALDH^{high} fraction. The CSC assay evaluating TIP has been critically regarded as a means to test the ability of tumor cells to evade or modulate the immune system, particularly T cell- and natural killer (NK) cell-mediated killing (Quintana et al., 2008). Therefore, to avoid potential confounding effects of the lack of an immune system, we performed the same assay in FVB/N mice. In fully immunocompetent mice, Lin$^-$CD90$^-$ALDH^{high} cells still have higher TIP ($p < 0.03$), although this is reduced to 2.7-fold compared with Lin$^-$CD90$^-$ALDH^{low} cells (Figure 1D). Furthermore, the difference in TIP compared with NSG grafts is reduced by 16.8-fold and 2.2-fold in Lin$^-$CD90$^-$ALDH^{high} and Lin$^-$CD90$^-$ALDH^{low}, respectively (Figure S1C). Next, we compared the tumor-initiation capacity of Lin$^-$CD24$^+$CD90$^+$ and Lin$^-$CD24$^+$CD90$^-$ cells. Our results show that Lin$^-$CD24$^+$CD90$^+$ cells have a decreased tumor-initiating ability in orthotopic limiting dilution assays ($p < 0.02$, Figures 1E and S1C). To test whether CD90$^-$ cells can give rise to CD90$^+$ cells, we took advantage of the allelic difference in CD90 between FVB/N (CD90.1) and NSG (CD90.2) mouse strains and were able to confirm that neither Lin$^-$ALDH^{high}CD90$^-$ nor Lin$^-$ALDH^{low}CD90$^-$ cells are able to give rise to Lin$^-$CD24$^+$CD90$^+$ *in vivo* (Figure 1F). However, CD90$^+$ tumors can give rise to CD90$^-$ cells (Figure 1G). As expected, CD90-depleted tumors show a 19-fold lower metastatic index when compared with CD90-containing tumors (Figure S1D). These results indicate that in the MMTV-PyMT model, CD90$^-$ tumor cells are lineage restricted but they harbor a strong TIP, while CD90$^+$ cancer cells retain a high metastatic potential.

CSC Populations Differ in Their Mesenchymal Traits

The mammosphere assay is frequently used to maintain stem cells *in vitro* and is often regarded as a surrogate for CSC content (Stingl et al., 2006). Spheres in the MMTV-PyMT model are composed of different cell types, including CD24$^+$CD90$^+$ cells (Figure 2A). To better characterize Lin$^-$CD24$^+$CD90$^+$ cells, we sorted them by FACS from tumors and performed qPCRs and cytospins, which showed that in the CD24$^+$ fraction CD90 is expressed in a population enriched in mesenchymal-like cells (Figures 2B–2D). Accordingly, Lin$^-$CD24$^+$CD90$^+$ cells FACS sorted from MMTV-PyMT tumors have little sphere-formation ability, while most of the sphere-formation capacity is found in the Lin$^-$CD90$^-$ALDH^{high} population (Figures 2E and S2). Interestingly, qPCR analyses on FACS-sorted Lin$^-$CD90$^-$ALDH^{high} and Lin$^-$CD90$^-$ALDH^{low} cells showed that the latter had a slightly more mesenchymal phenotype than Lin$^-$CD90$^-$ALDH^{high} cells (Figure 2F). Consistently, when we FACS sorted and grew both populations *in vitro*, Lin$^-$CD90$^-$ALDH^{low} cells showed a tendency to become more mesenchymal whereas Lin$^-$CD90$^-$ALDH^{high} cells formed epithelial colonies (Figure 2G). Taken together, these results indicate that in our model, sphere formation is associated with TIP-retaining epithelial progenitors.

Inhibition of TGFBR1 Produces More Sphere-Forming Cells

The acquisition of mesenchymal features through the EMT has been linked to the CSC phenotype (Mani et al., 2008). Since we had observed differences in epithelialization between metastatic CSCs and TICs, we next reasoned that blocking EMT might alter the proportions of CSCs in our system. Therefore, we treated MMTV-PyMT cells with a TGFBR1 inhibitor (SB431542). Surprisingly, treating the cells with the small molecule significantly increased sphere formation (Figure 3A). The same effects were achieved with the structurally different TGFBR1 inhibitor Ly2157299 (Figure S3A). In secondary sphere cultures, the tendency was maintained and the inhibitor still generated more spheres (Figure 3B). We observed similar results in other systems such as 4T1 and MMTV-Wnt1 cells (Figures S3B and S3C). Importantly, we also observed increased sphere formation in two out of three human breast cancer samples treated with SB431542 or Ly2157299 (Figures S3D–S3F). Conversely, adding TGFβ3 reduced sphere formation in all the models we tested (Figure S3). To investigate potential differences in the sensitivity to the inhibitor, we performed qPCR analyses on FACS-sorted MMTV-PyMT tumor cells using either the Lin$^-$CD24CD90 or the Lin$^-$CD90$^-$ALDH strategy. Interestingly, our results show that *Tgfbr1* expression is higher in Lin$^-$CD24$^+$CD90$^+$ and Lin$^-$CD90$^-$ALDH^{low} cells when compared with Lin$^-$CD24$^+$CD90$^-$ and Lin$^-$CD90$^-$ALDH^{high} cells, respectively (Figures 3C and 3D), which indicates that these subsets may be more sensitive to TGFBR1 inhibition. Furthermore, upon treating the cells with Noggin or transducing them with a secreted decoy receptor that acts as dominant negative form of the TGFBR2 (Zhao et al., 2002), we showed that the effects of the SB431542 on CSCs are mediated through TGFBR1/ALK5 and not ACVR1B/ALK4 or ACVR1C/ALK7 inhibition (Figures S3G and S3H; data not shown for Nodal). We next analyzed the proportion of ALDH^{high} cells upon treatment and found that TGFBR1

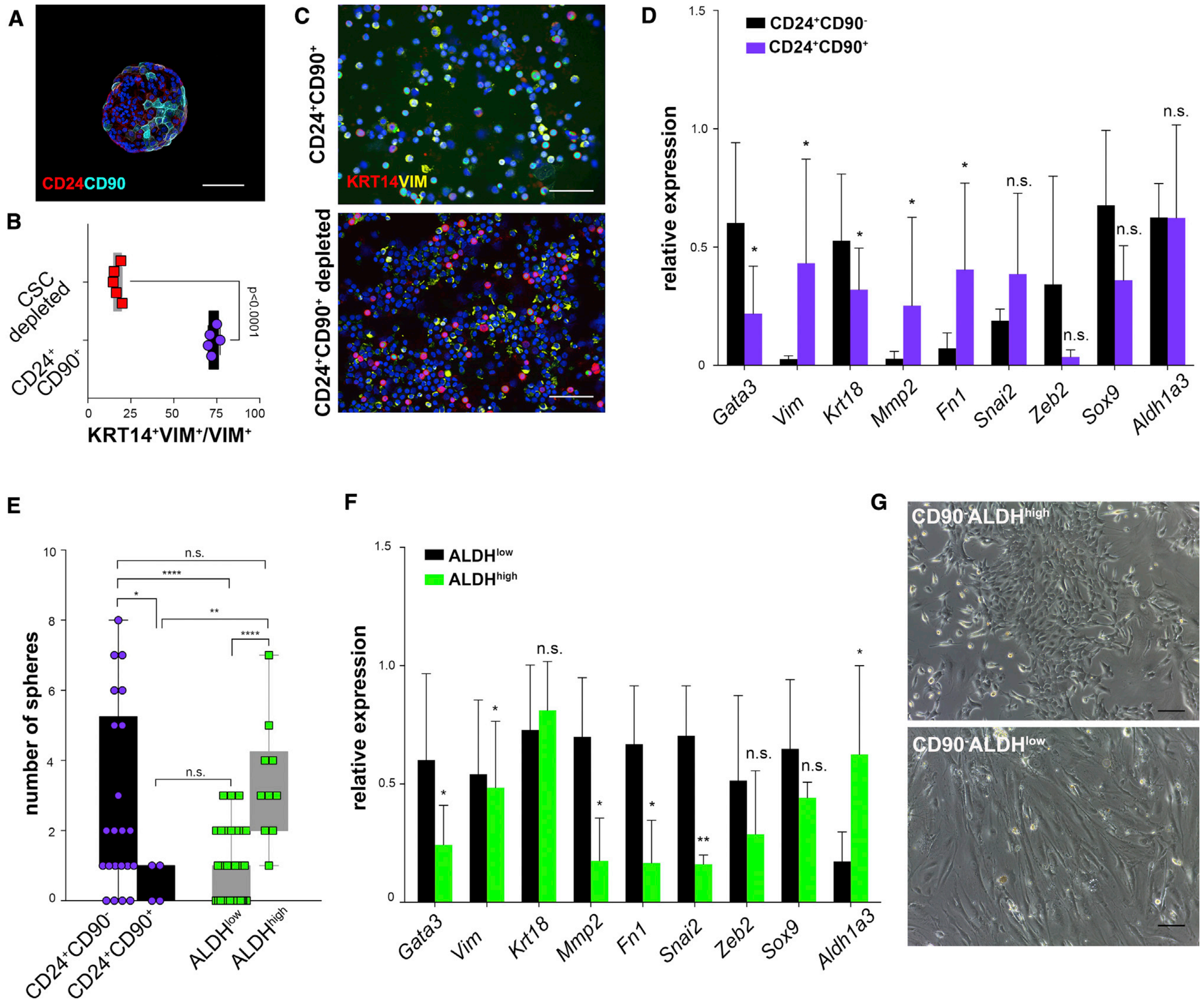

Figure 2. Characterization of CSCs

(A) Immunofluorescent analysis of mammospheres from the MMTV-PyMT model revealed that they contain a small fraction of $CD24^{+}CD90^{+}$ cells. Scale bar, 100 μm.

(B and C) FACS-sorted $Lin^{-}CD24^{+}CD90^{+}$ and $Lin^{-}CD24^{+}CD90^{+}$-depleted cells (B) were cytospinned, fixed, and stained for cytokeratin 14 and vimentin (C; scale bars, 100 μm). The number of double-positive cells in $vimentin^{+}$ cells was calculated using unpaired Student's t test (n = 5).

(D) qPCR on FACS-sorted $Lin^{-}CD24^{+}CD90^{+}$ and $Lin^{-}CD24^{+}CD90^{-}$ cells from fresh MMTV-PyMT tumors showed differences in gene expression (n = 6 independent tumors; *Rplp0* was used as a housekeeping gene; paired Student's t test).

(E) FACS sorting and culture of different populations of cells revealed that most of the sphere-formation ability is retained by $ALDH^{high}$ cells (n = 22 $CD24^{+}CD90^{-}$, n = 4 $CD24^{+}CD90^{+}$, n = 49 $ALDH^{low}$, n = 10 $ALDH^{high}$, for three independent tumors, one-way ANOVA and Fisher's LSD).

(F) qPCR on FACS-sorted $Lin^{-}CD90^{-}ALDH^{high}$ and $Lin^{-}CD90^{-}ALDH^{low}$ cells from fresh MMTV-PyMT tumors showed differences in gene expression (n = 4 independent tumors; *Rplp0* was used as a housekeeping gene; paired Student's t test).

(G) Culture of FACS-sorted $Lin^{-}CD90^{-}ALDH^{high}$ and $Lin^{-}CD90^{-}ALDH^{low}$ cells showed differences in morphology. Scale bars 100 μm.

*$p < 0.05$, **$p < 0.01$, ****$p < 0.0001$; n.s., not significant.

inhibition increased their percentage (Figure 3E), which is consistent with our previous results. In agreement with EMT causing a loss of TICs, treating the cells with TGFβ3 inhibited sphere formation, while this was rescued by addition of SB431542 (Figures 3F and S3I). Consistent with these observations, the inhibitor caused a significant

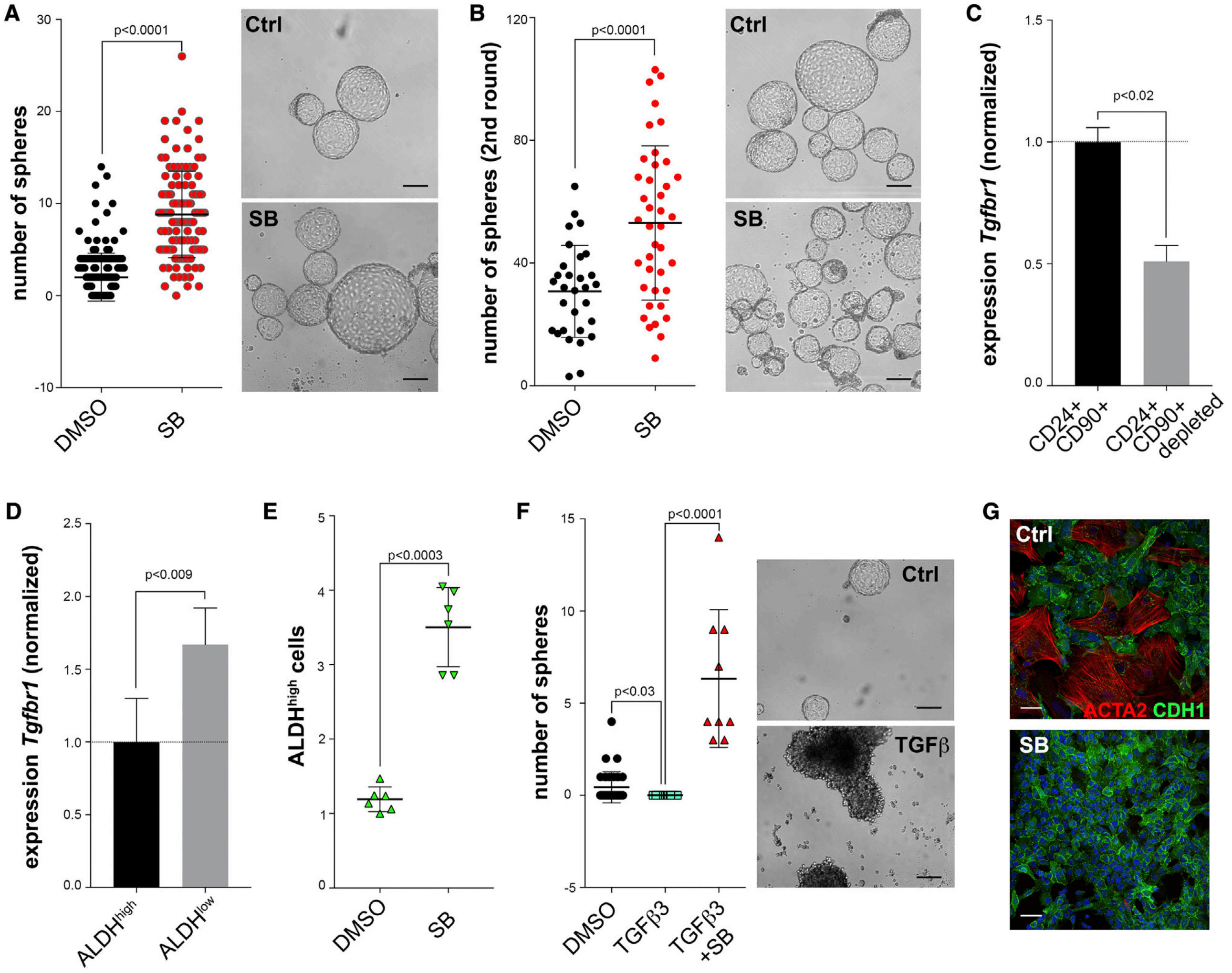

Figure 3. Inhibition of TGFBR1 Leads to an Increase in the Number of TICs

(A) Tumor cells were obtained from fresh MMTV-PyMT tumors, grown overnight in collagen-coated plates, and seeded as spheres (10^4 cells/well) in the presence of 2 μM SB431542 or dimethyl sulfoxide (DMSO). Spheres were counted 10 days later (n = 164 control and n = 122 SB, for six independent tumors; unpaired Student's t test). Scale bars, 100 μm.

(B) Primary spheres were trypsinized, counted, and replated in ultralow-attachment plates. Spheres were counted 10 days later (n = 30 not pretreated and n = 40 pretreated, for three independent experiments; unpaired Student's t test). Scale bars, 100 μm.

(C and D) qPCR analysis of *Tgfbr1* on FACS-sorted populations of MMTV-PyMT tumors using the CD24CD90 markers (C) or the AldeFluor assay (D) (n = 3 and n = 4 independent tumors, respectively, paired Student's t test).

(E) MMTV-PyMT tumors were digested and cells were plated and treated for 48 h with 2 μM SB431542 or DMSO. FACS analyses showed that TGFBR1 inhibition increased the frequency of $ALDH^{high}$ cells (n = 6 independent tumors, paired Student's t test).

(F) Tumor cells were obtained from fresh MMTV-PyMT tumors, grown overnight in collagen-coated plates, and seeded as spheres (10^4 cells/well) in the presence of DMSO, 1 ng/mL TGFβ3, or 1 ng/mL TGFβ3 and 2 μM SB431542. The number of spheres was determined after 10 days (n = 34 control, n = 20 TGFβ3, n = 9 TGFβ3+SB, for two independent tumors; one-way ANOVA and Fisher's LSD). Scale bars, 100 μm.

(G) Immunofluorescent staining for E-cadherin (CDH1) and α-smooth muscle actin (ACTA2) in cultured PyMT cells treated with 2 μM SB431542 or DMSO for 5 days. Scale bars, 50 μm.

reduction in mesenchymal traits as seen by immunostaining in attached cultures (Figure 3G). Overall, these results indicate that blocking TGFβ signaling through TGFBR1 inhibition is sufficient to trigger the expansion of tumor-initiating progenitors *in vitro*.

TGFβ Signaling Inhibition Reduces Metastasis but Not Tumor Initiation

We next produced three inducible short hairpin RNAs (shRNAs) for *Tgfbr1* (1,535, 825, and 777, Figure S4A) to validate our results *in vivo*. We infected PyMT cells with shTgfbr1

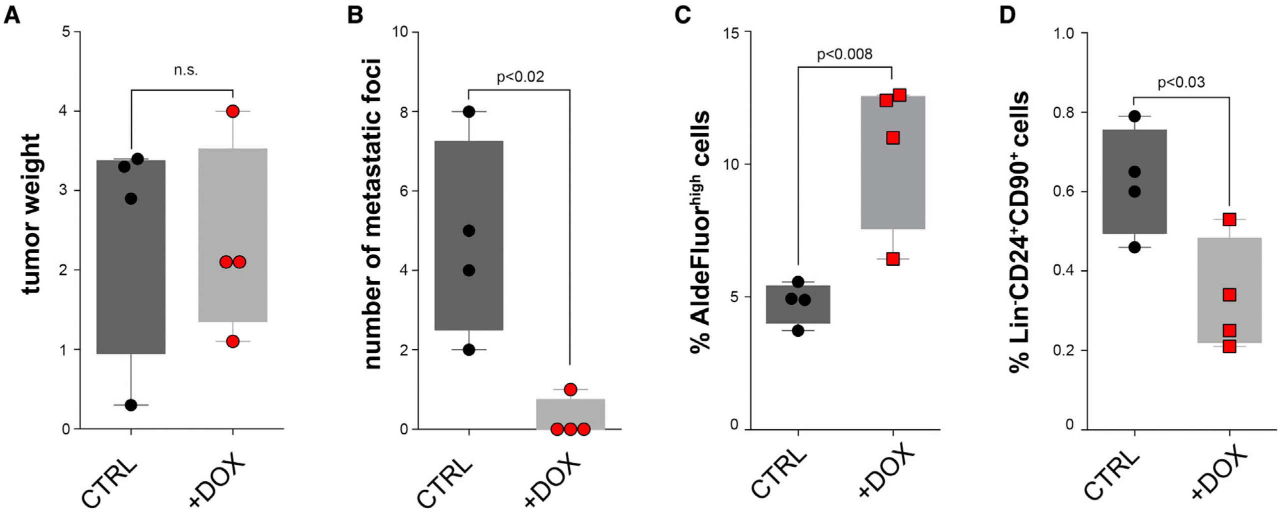

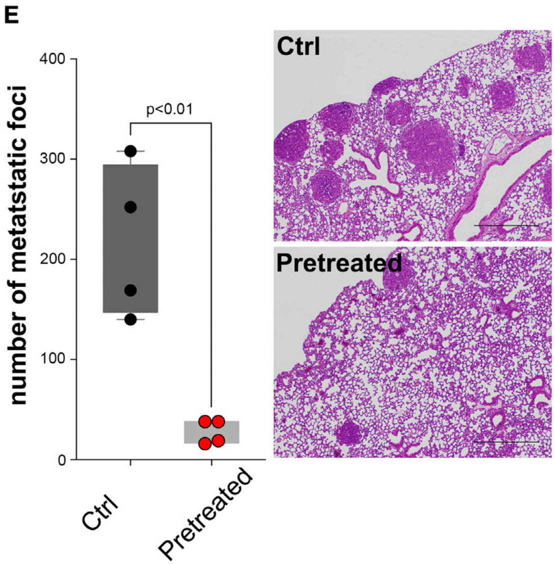

F

nº cells	DMSO	SB
500	4/8	5/6
50	0/8	3/8
5	0/8	0/8

Figure 4. Inhibition of TGFBR1 Decreases Metastasis but Increases Tumor-Initiation Potential

(A and B) MMTV-PyMT cells infected with a shTgfbr1 were grafted orthotopically into FVB/N mice, and one group was given doxycycline (DOX; 1 mg/mL) in the drinking water for the duration of the experiment. Both groups formed equally sized tumors (A), but downregulation of *Tgfbr1* led to a significant decrease in lung metastases (B) (n = 4, unpaired Student's t test).

(C and D) Tumors in which *Tgfbr1* was downregulated showed increased frequencies of $Lin^{-}ALDH^{high}$ cells (C; n = 4, unpaired Student's t test) but a reduced number of $Lin^{-}CD24^{+}CD90^{+}$ cells (D; n = 4, unpaired Student's t test).

(E and F) Cells from fresh MMTV-PyMT tumors were plated and pretreated with either 2 μM SB431542 or DMSO for 5 days and thereafter injected via tail vein or othotopically in limiting dilution assays in FVB/N mice. Pretreating the cells with SB431542 resulted in decreased metastatic colonization potential upon tail vein injection (E; n = 4, unpaired Student's t test; scale bars, 500 μm), while orthotopic injection in limiting dilution assays revealed that pretreated cells exhibit higher TIP (F).

lentiviruses and injected them orthotopically in FVB/N mice. While tumors did not differ in size, we observed a significant reduction in metastasis in those animals with tumors in which we downregulated *Tgfbr1* by doxycycline-induced shRNA expression (Figures 4A and 4B). Similar results were obtained using 4T1 cells in BALB/c mice (Figures S4B and S4C). Downregulating *Tgfbr1* in PyMT cells was consistently sufficient to increase the proportion of $Lin^{-}ALDH^{high}$ cells and reduce that of $Lin^{-}CD24^{+}CD90^{+}$ *in vivo* (Figures 4C and 4D, respectively). Finally, we treated PyMT cells with SB431542 and injected them either via tail vein or orthotopically in limiting dilution assays. Cells that were pretreated showed reduced metastatic ability in lung metastasis assays (Figure 4E), but had higher TIP (Figure 4F,

$p < 0.03$). Of note, SB431542 does not affect cell viability *in vitro* (Figure S4D). Taken together, these results indicate that TGFβ signaling regulates metastatic and tumor-initiating CSC.

DISCUSSION

CSCs are defined as a subset of cells in a tumor, which possess stem cell properties and sustain tumor growth. These properties are inherent to some particular cells within the tumor, but can also be acquired, which reflects a certain degree of cellular plasticity (Batlle and Clevers, 2017). In agreement with this rather broad definition, different subsets of CSCs have been identified in different tumors including breast (Yeo et al., 2016). Furthermore, plasticity in the stem cell niche is known to be an important factor regulating transitions to different stem-like states in CSCs (Brooks et al., 2015). The CSC field suffers from a major drawback, namely the lack of universal markers: they can vary from mouse to human and between different models of the same type of cancer. Ginestier et al. (2007) showed that in human breast cancer, the overlap between ALDH^{high} and Lin$^-$CD44$^+$CD24$^-$ cells is small (1.16%), and that most of the TIP is retained by the ALDH^{high}non(CD24$^-$CD44$^+$) and ALDH^{high}CD24$^-$CD44$^+$ fractions, while ALDH^{low} CD24$^-$CD44$^+$ have little TIP. Nevertheless, this isolation strategy (CD24CD44) cannot be formally compared with ours (CD24CD90), because it is not known whether CD44$^+$CD24$^-$ are bona fide metastatic stem cells. Likewise, whether the presence/abundance of CD24$^-$CD44$^+$ predicts poor prognosis or is associated with distant metastasis is a matter of debate (Abraham et al., 2005; Mylona et al., 2008; Nogi et al., 2011; Wei et al., 2012). We here used the MMTV-PyMT model to show that two populations of tumor cells with distinct tumor-initiating abilities coexist within mammary tumors. The first, which we described as retaining most metastatic potential in the tail vein injection assay, has mesenchymal features and is defined as Lin$^-$CD24$^+$CD90$^+$ (Malanchi et al., 2012). In addition, we now found the lineage-restricted Lin$^-$CD90$^-$ALDH^{high} epithelial-like population to be highly enriched in cells with TIP but with poorer metastasis-initiating capacity. As expected, grafting the same tumor cells in NSG or FVB mice produced significant differences in the estimated stem cell frequency. When grafted in NSG mice, Lin$^-$CD90$^-$ALDH^{high} cells had an estimated 14.3% of TICs while in FVB, the figure dropped to 0.8%. These results suggest that in the MMTV-PyMT model, approximately 94% of the tumor cells showing tumor-initiation capacity when grafted in NSG mice are killed by T or NK cells when grafted into immunocompetent mice, and therefore cannot be considered bona fide CSCs (Bruttel and Wischhusen, 2014). This issue has been observed by a number of groups and underlines the importance of using syngeneic models in immunocompetent mice (Quintana et al., 2008).

To understand how these subsets of CSCs are controlled, we modulated TGFβ signaling and found that it shifts the proportions of these two CSC populations. Not surprisingly, blocking TGFβ signaling inhibits the metastatic population and interferes with the metastatic cascade. However, it also triggers the expansion of ALDH^{high} cells, which have a high tumor-initiation capacity. Our results are in contrast to those previously published showing that in pancreatic cancer TGFβ inhibition decreases CSC numbers, including sphere-forming cells (Donahue and Dawson, 2011). However, it is well known that in breast cancer TGFβ plays pleiotropic roles that are context dependent and, therefore, this may be one of the reasons explaining the differences observed (David and Massague, 2018; Fang et al., 2013). Bhola et al. (2013) showed that in combination with paclitaxel, TGFβ inhibition decreased the frequency of triple-negative breast cancer TICs. Interestingly, their data indicate that treating SUM159 cells with the small molecule Ly2157299 increased ALDH$^+$ cells and sphere formation, both surrogates for TIP, which is in agreement with our results. In a recent work, Beerling et al. (2016) suggested that cellular plasticity uncouples the effects of EMT on CSCs. Our results show that, in our model, secondary and primary TIP are features not necessarily shared by the same cell type that can be regulated by TGFBR1 inhibition. These data indicate that TGFBR1 inhibitors might exert different effects that are cell and context dependent. These results may have important implications for treatment, since TGFβ inhibitors are currently under clinical trials. In particular, it might be relevant for those patients with circulating tumor cells (CTCs). The presence of CTCs in patients with breast cancer is associated with bad prognosis (Cristofanilli et al., 2005). It was recently shown that metastases can be polyclonal, i.e., arising from multiple clones that seed the secondary site (Cheung et al., 2016). This is in line with previous results suggesting that CTC clusters are the precursors of polyclonal metastases (Aceto et al., 2014). Our data suggest the possibility that treatment with TGFβ inhibitors might promote the expansion of circulating tumor stem-like cell clusters, and therefore advise caution when using them to treat patients with breast cancer.

EXPERIMENTAL PROCEDURES

Detailed methods for FACS, qPCR, western blot analysis, and cell culture are provided in Supplemental Information.

Mouse Work

MMTV-PyMT (FVB/N) mice were bred and housed in ventilated cages in the OHB mouse husbandry of the University of Fribourg.

For PyMT tumor cell transplantation to the fourth mammary fat pad or tail vein injection experiments, we used NSG and immunocompetent FVB/N mice. The experiments involving 4T1 cell injections were done in immunocompetent BALB/c mice. For limiting dilution experiments, cells were injected in Matrigel/PBS (1:3). All the experimental procedures involving mice were carried out in accordance with the Swiss Animal Welfare Regulations and were previously approved by the Cantonal Veterinary Service of the Canton Fribourg (2017_26_FR).

Statistics

The results were analyzed using GraphPad Prism 7 software. Means were compared with either paired or unpaired Student's t test. In case groups would not pass a normality test (assessed using D'Agostino-Pearson's omnibus normality test), samples were analyzed with the Mann-Whitney non-parametric test. When comparing more than two variables, we performed one-way ANOVA. To isolate differences between groups, we performed Fisher's least significant difference (LSD) test. p values are indicated for each experiment. Limiting dilution assay data were analyzed using ELDA (extreme limiting dilution assay) (Hu and Smyth, 2009). Experiments were done at least in triplicate. Error bars indicate standard deviation. Significant differences between experimental groups are indicated with asterisks in the figures as follows: *$p < 0.05$, **$p < 0.01$, ***$p < 0.001$, and ****$p < 0.0001$.

SUPPLEMENTAL INFORMATION

Supplemental Information can be found online at https://doi.org/10.1016/j.stemcr.2019.05.026.

AUTHOR CONTRIBUTIONS

Conceptualization, A.S.-M.; Methodology, A.S.-M. and F.F.; Investigation, F.F., A.S.-M., and M.B.; Formal Analysis, F.F. and A.S.-M.; Resources, A.S.-M. and C.R.; Writing – Original Draft, A.S.-M. and F.F.; Writing – Review & Editing, A.S.-M. and F.F.; Funding Acquisition, A.S.-M.; Supervision, A.S.-M. All authors read and approved the final manuscript.

ACKNOWLEDGMENTS

The authors are grateful to Prof. Joerg Huelsken for insightful scientific discussions, Dr. Paloma Ordóñez-Moran for kindly providing the Noggin, Ms. Vanina Lauper and Ms. Nancy Thompson for excellent technical assistance, Ms. Mariana Clar for her contribution in the lab, and Dr. Hans-Anton Lehr for organizing the collection of human samples. A.S.-M. and F.F. are deeply grateful to the Swiss National Science Foundation (SNSF) and the Swiss Cancer League for supporting our research. This project was supported by an SNSF Ambizione career award to A.S.-M. (PZ00P3_154751). F.F. was supported by the Swiss Cancer League grant KLS-4121-02-2017 to A.S.-M. M.B. was supported by the SNSF Sinergia grant CRSII3_154499/1 to C.R. The authors declare that they have no competing interests.

Received: October 18, 2018
Revised: May 27, 2019
Accepted: May 27, 2019
Published: June 27, 2019

REFERENCES

Abraham, B.K., Fritz, P., McClellan, M., Hauptvogel, P., Athelogou, M., and Brauch, H. (2005). Prevalence of CD44^{+}/CD24^{-}/low cells in breast cancer may not be associated with clinical outcome but may favor distant metastasis. Clin. Cancer Res. *11*, 1154–1159.

Aceto, N., Bardia, A., Miyamoto, D.T., Donaldson, M.C., Wittner, B.S., Spencer, J.A., Yu, M., Pely, A., Engstrom, A., Zhu, H., et al. (2014). Circulating tumor cell clusters are oligoclonal precursors of breast cancer metastasis. Cell *158*, 1110–1122.

Batlle, E., and Clevers, H. (2017). Cancer stem cells revisited. Nat. Med. *23*, 1124–1134.

Beerling, E., Seinstra, D., de Wit, E., Kester, L., van der Velden, D., Maynard, C., Schafer, R., van Diest, P., Voest, E., van Oudenaarden, A., et al. (2016). Plasticity between epithelial and mesenchymal states unlinks EMT from metastasis-enhancing stem cell capacity. Cell Rep. *14*, 2281–2288.

Bhola, N.E., Balko, J.M., Dugger, T.C., Kuba, M.G., Sanchez, V., Sanders, M., Stanford, J., Cook, R.S., and Arteaga, C.L. (2013). TGF-beta inhibition enhances chemotherapy action against triple-negative breast cancer. J. Clin. Invest. *123*, 1348–1358.

Brooks, M.D., Burness, M.L., and Wicha, M.S. (2015). Therapeutic implications of cellular heterogeneity and plasticity in breast cancer. Cell Stem Cell *17*, 260–271.

Bruttel, V.S., and Wischhusen, J. (2014). Cancer stem cell immunology: key to understanding tumorigenesis and tumor immune escape? Front. Immunol. *5*, 360.

Celia-Terrassa, T., Meca-Cortes, O., Mateo, F., de Paz, A.M., Rubio, N., Arnal-Estape, A., Ell, B.J., Bermudo, R., Diaz, A., Guerra-Rebollo, M., et al. (2012). Epithelial-mesenchymal transition can suppress major attributes of human epithelial tumor-initiating cells. J. Clin. Invest. *122*, 1849–1868.

Cheung, K.J., Padmanaban, V., Silvestri, V., Schipper, K., Cohen, J.D., Fairchild, A.N., Gorin, M.A., Verdone, J.E., Pienta, K.J., Bader, J.S., et al. (2016). Polyclonal breast cancer metastases arise from collective dissemination of keratin 14-expressing tumor cell clusters. Proc. Natl. Acad. Sci. U S A *113*, E854–E863.

Cristofanilli, M., Hayes, D.F., Budd, G.T., Ellis, M.J., Stopeck, A., Reuben, J.M., Doyle, G.V., Matera, J., Allard, W.J., Miller, M.C., et al. (2005). Circulating tumor cells: a novel prognostic factor for newly diagnosed metastatic breast cancer. J. Clin. Oncol. *23*, 1420–1430.

David, C.J., and Massague, J. (2018). Contextual determinants of TGFbeta action in development, immunity and cancer. Nat. Rev. Mol. Cell Biol. *19*, 419–435.

Donahue, T.R., and Dawson, D.W. (2011). Nodal/Activin signaling: a novel target for pancreatic cancer stem cell therapy. Cell Stem Cell *9*, 383–384.

Fang, Y., Chen, Y., Yu, L., Zheng, C., Qi, Y., Li, Z., Yang, Z., Zhang, Y., Shi, T., Luo, J., et al. (2013). Inhibition of breast cancer metastases by a novel inhibitor of TGFbeta receptor 1. J. Natl. Cancer Inst. *105*, 47–58.

Ginestier, C., Hur, M.H., Charafe-Jauffret, E., Monville, F., Dutcher, J., Brown, M., Jacquemier, J., Viens, P., Kleer, C.G., Liu, S., et al. (2007). ALDH1 is a marker of normal and malignant human

mammary stem cells and a predictor of poor clinical outcome. Cell Stem Cell *1*, 555–567.

Hu, Y., and Smyth, G.K. (2009). ELDA: extreme limiting dilution analysis for comparing depleted and enriched populations in stem cell and other assays. J. Immunol. Methods *347*, 70–78.

Liu, S., Cong, Y., Wang, D., Sun, Y., Deng, L., Liu, Y., Martin-Trevino, R., Shang, L., McDermott, S.P., Landis, M.D., et al. (2014). Breast cancer stem cells transition between epithelial and mesenchymal states reflective of their normal counterparts. Stem Cell Reports *2*, 78–91.

Malanchi, I., Santamaria-Martinez, A., Susanto, E., Peng, H., Lehr, H.A., Delaloye, J.F., and Huelsken, J. (2012). Interactions between cancer stem cells and their niche govern metastatic colonization. Nature *481*, 85–89.

Mani, S.A., Guo, W., Liao, M.J., Eaton, E.N., Ayyanan, A., Zhou, A.Y., Brooks, M., Reinhard, F., Zhang, C.C., Shipitsin, M., et al. (2008). The epithelial-mesenchymal transition generates cells with properties of stem cells. Cell *133*, 704–715.

Mylona, E., Giannopoulou, I., Fasomytakis, E., Nomikos, A., Magkou, C., Bakarakos, P., and Nakopoulou, L. (2008). The clinicopathologic and prognostic significance of $CD44^{+}/CD24^{(-/low)}$ and $CD44^{-}/CD24^{+}$ tumor cells in invasive breast carcinomas. Hum. Pathol. *39*, 1096–1102.

Nogi, H., Suzuki, M., Kamio, M., Kato, K., Kawase, K., Toriumi, Y., Takeyama, H., Fukushima, H., Morikawa, T., and Uchida, K. (2011). Impact of $CD44^{+}CD24^{-}$ cells on non-sentinel axillary lymph node metastases in sentinel node-positive breast cancer. Oncol. Rep. *25*, 1109–1115.

Ocana, O.H., Corcoles, R., Fabra, A., Moreno-Bueno, G., Acloque, H., Vega, S., Barrallo-Gimeno, A., Cano, A., and Nieto, M.A. (2012). Metastatic colonization requires the repression of the epithelial-mesenchymal transition inducer Prrx1. Cancer Cell *22*, 709–724.

Quintana, E., Shackleton, M., Sabel, M.S., Fullen, D.R., Johnson, T.M., and Morrison, S.J. (2008). Efficient tumour formation by single human melanoma cells. Nature *456*, 593–598.

Stingl, J., Eirew, P., Ricketson, I., Shackleton, M., Vaillant, F., Choi, D., Li, H.I., and Eaves, C.J. (2006). Purification and unique properties of mammary epithelial stem cells. Nature *439*, 993–997.

Tsai, J.H., Donaher, J.L., Murphy, D.A., Chau, S., and Yang, J. (2012). Spatiotemporal regulation of epithelial-mesenchymal transition is essential for squamous cell carcinoma metastasis. Cancer Cell *22*, 725–736.

Wan, L., Lu, X., Yuan, S., Wei, Y., Guo, F., Shen, M., Yuan, M., Chakrabarti, R., Hua, Y., Smith, H.A., et al. (2014). MTDH-SND1 interaction is crucial for expansion and activity of tumor-initiating cells in diverse oncogene- and carcinogen-induced mammary tumors. Cancer Cell *26*, 92–105.

Wei, W., Hu, H., Tan, H., Chow, L.W., Yip, A.Y., and Loo, W.T. (2012). Relationship of $CD44^{+}CD24^{-/low}$ breast cancer stem cells and axillary lymph node metastasis. J. Transl. Med. *10* (*Suppl 1*), S6.

Yeo, S.K., Wen, J., Chen, S., and Guan, J.L. (2016). Autophagy differentially regulates distinct breast cancer stem-like cells in murine models via EGFR/Stat3 and TGFbeta/Smad signaling. Cancer Res. *76*, 3397–3410.

Zhao, W., Kobayashi, M., Ding, W., Yuan, L., Seth, P., Cornain, S., Wang, J., Okada, F., and Hosokawa, M. (2002). Suppression of in vivo tumorigenicity of rat hepatoma cell line KDH-8 cells by soluble TGF-beta receptor type II. Cancer Immunol. Immunother. *51*, 381–388.

Stem Cell Reports, Volume *13*

Supplemental Information

Breast Cancer Stem Cells with Tumor- versus Metastasis-Initiating Capacities Are Modulated by TGFBR1 Inhibition

Flavia Fico, Mélanie Bousquenaud, Curzio Rüegg, and Albert Santamaria-Martínez

Inventory of Supplemental Information

Supplemental Figure 1. Metastatic stem cells vs tumor-initiating cells. Related to Figure 1.

Supplemental Figure 2. Characterization of CSC. Related to Figure 2.

Supplemental Figure 3. Inhibition of TGFBR1 leads to an increase in the number of TIC. Related to Figure 3.

Supplemental Figure 4. Inhibition of TGFBR1 decreases metastasis but increases TIP. Related to Figure 4.

Supplemental Experimental Procedures

- Antibodies and reagents
- Mouse work extended
- Orthotopic transplants
- Tail vein injections
- Cell culture
- Tumor sphere assays
- Lentiviral production
- FACS analysis
- Immunostaining
- Western blot
- Real-time PCR
- MTT assay

Supplemental File 5. Table 1: Primer sets used in this study.

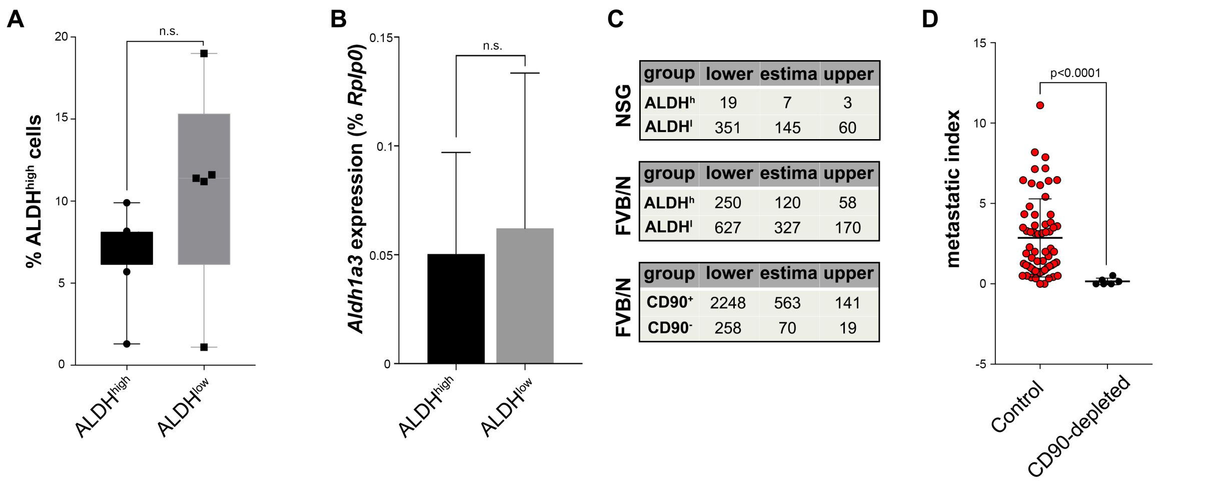

Supplemental Figure 1. Metastatic stem cells vs tumor-initiating cells. Related to Figure 1. (A) Tumors generated using FACS-sorted ALDH^{high} and ALDH^{low} MMTV-PyMT cells were digested and re-analysed again using the AldeFluor assay (n=5 independent tumors, Mann-Whitney test). (B) Tumors generated using FACS-sorted ALDH^{high} and ALDH^{low} were pulverised in liquid nitrogen, RNA was extracted and qPCRs were performed to evaluate the expression of *Aldh1a3* transcripts (n=5 independent tumors, unpaired Student's t test). (C) Cancer stem cell frequency with confidence intervals of ALDH^{high} and ALDH^{low} cells or CD24^{+}CD90^{+}/CD24^{+}CD90^{-} shown in Figure 1B, 1D and 1E. (D) Metastatic index of CD90-depleted tumors vs CD90^{+} cells containing tumors was calculated as the tumor weight (g) divided by the number of metastatic foci in the lungs (n=62 control and n=6 CD90-depleted independent tumors, Mann-Whitney test).

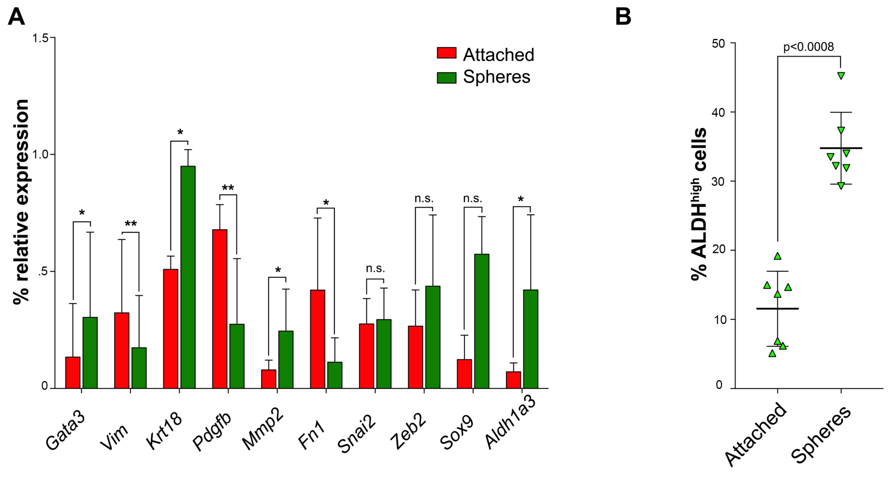

Supplemental Figure 2. Characterization of CSC. Related to Figure 2. (A) qPCR comparing attached vs sphere cultures of MMTV-PyMT cells derived from the same tumors (n=8 independent tumors, paired Student's t test). (B) MMTV-PyMT tumors were digested and cells were plated either in 2D or 3D cultures. FACS analyses show that tumors plated as spheres have a higher frequency of ALDH^{high} cells (n=7 independent tumors, paired Student's t test).

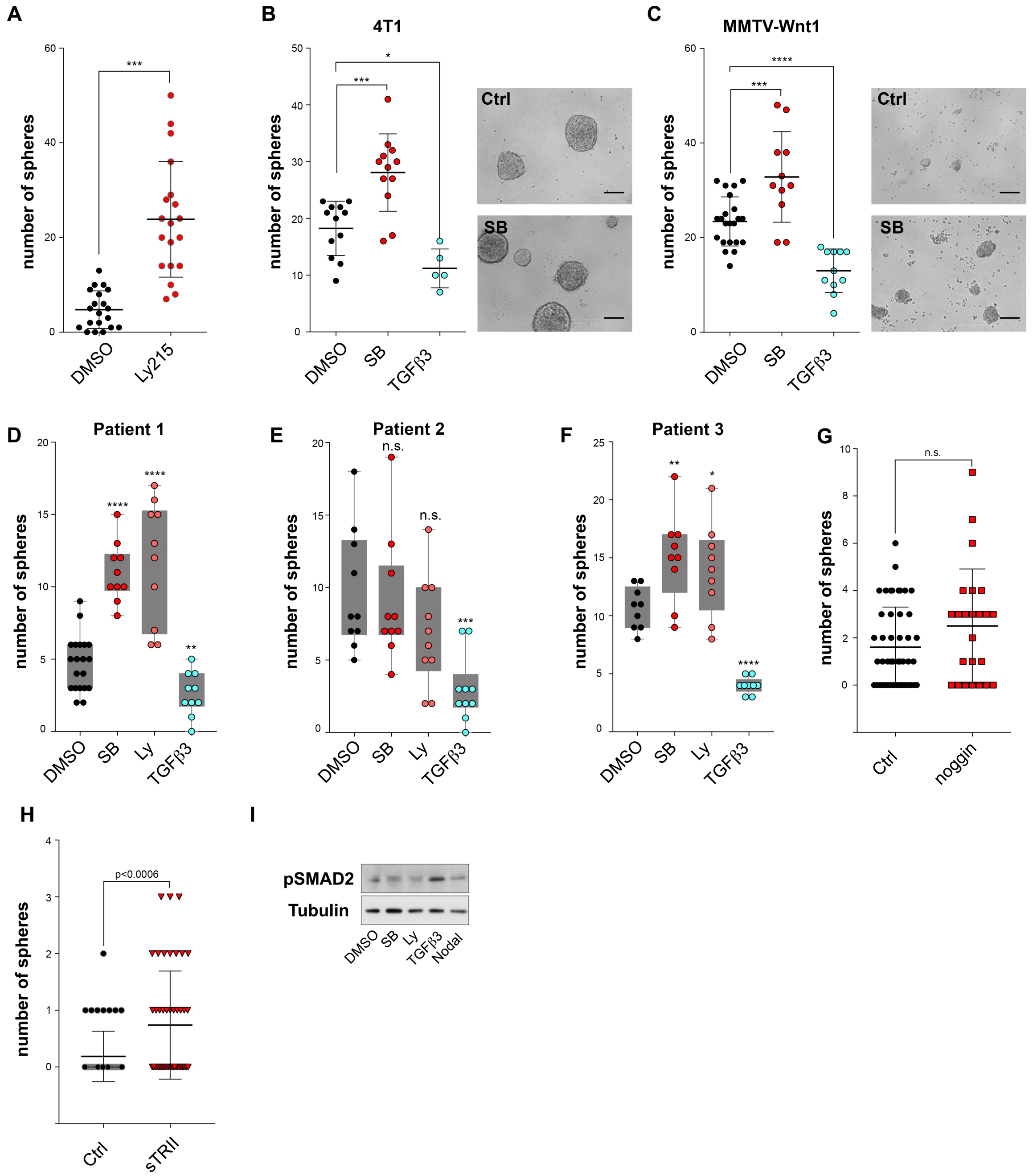

Supplemental Figure 3. Inhibition of TGFBR1 leads to an increase in the number of TIC. Related to Figure 3. (A) Tumor cells were obtained from fresh MMTV-PyMT tumors, grown overnight in collagen-coated plates and then seeded as spheres (10^4 cells/well) in presence of either 2 µM Ly2157299 or DMSO. Spheres were counted 10 days later (n=20 DMSO, n=19 Ly, for 3 independent tumors, unpaired Student's t test). (B, C) 4T1 (n= 12 DMSO, n=12 SB, n=5 TGFβ3) and MMTV-Wnt1 cells (n=22 DMSO, n=11 SB, n=11 TGFβ3) were plated as spheres (10^4 or $5x10^3$ cells/well and 10^4 cells/well, respectively) and treated with 2 µM SB431542, 1 ng/ml TGFβ3 or DMSO. Spheres were counted 7-10 days later (One-way ANOVA and Fisher's LSD, scale bar 100 µm). (D-F) Human breast cancer tissue was digested with collagenase and seeded directly in ultralow attachment plates with media containing DMSO, 2 µM SB431542, 2 µM Ly2157299 or 1 ng/ml TGFβ3. Spheres were counted one week later (One-way ANOVA and Fisher's LSD). (G) Tumor cells were obtained from fresh MMTV-PyMT tumors, grown overnight in collagen-coated plates and then seeded as spheres (10^4 cells/well) in presence or absence of 500 ng of noggin. Spheres were counted 10 days later (n=48 DMSO, n=24 noggin, for 4 independent tumors, unpaired Student's t test). (H) Tumor cells were obtained from fresh MMTV-PyMT tumors, grown overnight in collagen-coated plates, trypsinized and infected with lentiviruses containing either the mock or a TGFBR2 decoy receptor (sTRII), selected and seeded in ULA plates. Spheres were counted 10 days later (n=48 ctrl, n=46 sTRII, for 2 independent tumors, unpaired Student's t test). (I) Spheres were collected by centrifugation and protein extracts were subjected to Western blot analysis of pSMAD2 to test the TGFβ pathway activation.

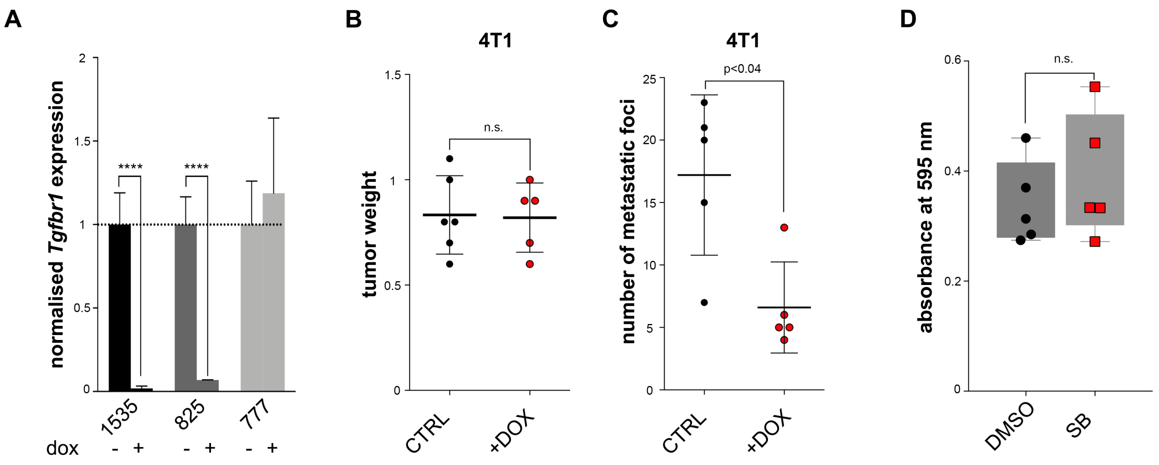

Supplemental Figure 4. Inhibition of TGFBR1 decreases metastasis but increases tumor initiation potential. Related to Figure 4. (A) Fresh MMTV-PyMT tumor-derived cells were infected in suspension with lentiviruses containing the *Tgfbr1* short hairpins. The expression was induced treating the cells with with 1µg/ml doxycycline for 48h. The graphs show the normalized mRNA expression levels for *Tgfbr1* in activated vs non-activated cells. (B, C) 4T1 cells infected with a shTgfbr1 were grafted orthotopically into BALB/c mice and one group was given doxycycline (1 mg/ml) in the drinking water for the duration of the experiment. Both groups formed equally sized tumors, but downregulation of *Tgfbr1* led to a significant decrease in lung metastases (n=5, unpaired Student's t test). (D) MMTV-PyMT cells were assayed for MTT upon treatment with either 2 µM SB431542 or DMSO (n=5 independent tumors, paired Student's t test).

Supplemental Experimental Procedures

Antibodies and reagents

CD90.1 (HIS51), CD90.2 (53-2.1), CD24 (M1/69), eBioscience; Ter119, CD31 (MEC13.3), CD45 (30-F11), Biolegend; Cytokeratin 14 (Covance); pSMAD2, E-Cadherin (Cell Signaling); α-SMA, β-Tubulin (Sigma); Vimentin (Lifespan Biosciences); SB431542 (Stem Cell Technologies); Ly2157299 (Selleckchem), TGFβ (Promega). Both small molecules were dissolved using DMSO and stocked at 10 mM. The plasmids containing the short hairpins for Tgfbr1 were produced by classical molecular cloning and subcloned into pRRL vectors containing a doxycycline inducible *tet* system and a blasticidin resistance gene for mammalian selection. The sequences of the hairpins are (5'-3'): Alk5_1535: TTAATTCGCAAAGCTGTCAGCC; Alk5_777: AGAAGAGAATATCTTCACAGCA; Alk5_825: TACAGTCTGATAAATCTCTGCC.

Mouse work extended

Orthotopic transplants

To generate orthotopic transplants of MMTV-PyMT tumor cells, FVB/N mice were injected with 10^6 PyMT cells resuspended in 50 μl of PBS in the 4th mammary fat pad using an insulin syringe (26G). 4T1 tumors were generated by injecting $2.5x10^5$ cells in the 4th mammary fat pad of BALB/c mice using an insulin syringe (26G). Metastatic foci in the lungs were counted using a Leica M125 stereomicroscope.

Tail vein injections

Mice were warmed by placing the cage under an IR light bulb. One mouse at a time was placed in a tube rodent holder for tail vein injection, with the tail outside of the tube. The tail was cleaned with 70% ethanol. The IR light bulb was placed above the tail to cause the veins to dilate. MMTV-PyMT tumor cells ($5x10^5$ per mouse) were resuspended in PBS and injected very slowly in a 100 μl volume into one of the two tail veins using an insulin syringe (26G needle). The spot of injection was then compressed with a tissue to make sure the tail was not bleeding. Mice were returned to the cage and kept for observation for 15 min. Metastatic foci in the lungs were counted after 3-5 weeks using a Leica M125 stereomicroscope.

Cell culture

Mouse tumor tissue was dissociated mechanically, followed by an incubation with 1:66 Liberase TH (Roche) and DNAse (10 mg/ml) at 37ºC for 1 hour. Cells were then washed twice in 2 mM EDTA in PBS and once in PBS and then plated in collagen-coated plates (HBSS, BSA 100 mg/ml, HEPES 1M pH 6.5 and bovine collagen biomatrix by Cell Systems). Cells were grown in DMEM:F12 (Gibco) supplemented with 2% FBS, 1% penicillin/streptomycin 20 ng/ml EGF (Invitrogen) and 10 μg/ml insulin (Invitrogen) and let attach overnight. 4T1 cells were obtained from the ATCC and grown as recommended. Human breast tumor tissue was obtained under informed consent from patients undergoing breast surgery (tumorectomy or mastectomy) for an untreated breast cancer. Tissues were collected in HBSS+2% FBS+1% penicillin/streptomycin. They were then digested mechanically and enzymatically with a 0.3% collagenase IV (Sigma) containing solution for 1 hour at 37ºC.

Tumor sphere assays

Sphere cultures were established from fresh total tumor cell preparations. After dissociation, tumor cells were plated on collagen-coated plates overnight, trypsinized next day and plated in 150 μl of sphere media (DMEM/F12 with B27, 20 ng/ml EGF, 20 ng/ml FGF, 4 μg/ml heparin, 1% penicillin/streptomycin) with or without the small molecules at the indicated concentrations into 96-well low attachment plates (Cornig) at $1x10^4$ cells per well and at least 5 wells per tumor. MMTV-PyMT spheres were counted after one week (7-10 days). Secondary mammosphere culture was performed by collecting the spheres through gentle centrifugation (800 rpm) followed by enzymatic (10 min in 0.05% trypsin-EDTA at 37°C) mechanical dissociation using a pipette. Mammospheres were stained with the indicated antibodies and images were taken with an inverted confocal microscope.

Lentiviral production

Lentiviral particles were produced in HEK293T cells by calcium phosphate precipitation. Briefly, two hours prior to transfection, $11x10^6$ HEK293T cells/15 cm dish were incubated with DMEM+10%FBS supplemented with 25 μM chloroquine (Sigma). Lentiviruses were produced by cotransfection of HEK293T cells with the vectors of interest together with the pCMV-dR8.74 and the pMD2G (VSVG). Next day, media was removed and replaced with fresh media containing 3 mM caffeine (Sigma). On the third day, the supernatant containing the viral particles was collected, ultracentrifuged for 2.5 hours at 20000 rpm to concentrate the lentiviruses and used to infect cells or aliquoted and stored at -80ºC.

FACS Analysis

For FACSorting experiments, tumor cells derived from MMTV-PyMT mice were obtained by enzymatic disaggregation as described above. Cells were then washed twice with PBS, filtered (0.70 µM filters), stained with the appropriate antibodies for 30 minutes at 4ºC and sorted using either a FACSAria, a FACSAria III (BD Biosciences) or a MoFlo Astrios (Beckman Coulter). For FACS analysis, tumor cells were trypsinized, washed and stained with the appropriate antibodies for 30 minutes at 4ºC. DAPI or 7-AAD were used to discard dead cells. ALDH activity was tested using the AldeFluor^{TM} assay kit (Stem Cell Technologies) as per the manufacturer's protocol. Briefly, cells were incubated with either the AldeFluor^{TM} reagent alone or the AldeFluor^{TM} reagent and the inhibitor diethylaminobenzaldehyde (DEAB) for 30 minutes at 37ºC. Cells were then centrifuged, washed and immunophenotyped when required. Fluorescence was analysed using either a Cyan ADP (Dako) or a MACSQuant (Miltenyi) instrument. Data was processed and analysed using FlowJo.

Immunostaining

MMTV-PyMT cells were grown on glass cover-slips, treated with vehicle (DMSO) or 2 µM SB431542 for 5 days and then fixed with 4% PFA for 15 minutes and subsequently washed with PBS. The unspecific binding of the antibodies was blocked using a solution containing 10% serum for 1 hour at room temperature. Samples were then incubated with primary antibodies for 1 hour at room temperature, washed 3 times with 1x PBS and incubated with a secondary antibody conjugated to AlexaFluor-488 for 1 hour at room temperature in a humid-chamber protected from light. Cell nuclei were stained with DAPI. Fluorescent images were taken with a LSM700 inverted confocal microscope (Zeiss), setting the pinhole at 1 AU.

Western blot

Protein was extracted with complete RIPA buffer (20 mM Tris-HCl (pH 7.5), 150 mM NaCl, 1 mM Na2EDTA, 1 mM EGTA, 1% NP-40, 1% sodium deoxycholate, 2.5 mM sodium pyrophosphate, 1 mM β-glycerophosphate, 1 mM Na3VO4, 1 µg/ml leupeptin; Cell Signaling), separated by electrophoresis, transferred to PVDF membranes (Millipore), blocked with 5% BSA (Carl Roth) in 0.1% Tween 20 containing Tris-buffered saline (TBST) and incubated overnight with primary antibodies. Immunoreactive bands were visualized using HRP-conjugated secondary antibodies (Cell Signaling and Dako).

Real-time PCR

RNA was prepared using the mini or micro RNA kit (Qiagen) as per the manufacturer's instructions. cDNAs were generated using oligo-T priming and the M-MLV Reverse Transcriptase RNase H (-) Point Mutant (Promega). qPCR was performed in a StepOnePlus thermocycler (Applied Biosystems) using the SYBR green PCR Master Mix (Kapa) and following the manufacturer's instructions. A list of primers used is shown in Supplemental File 5: Table 1.

MTT assay

MTT [3-(4,5-Dimethylthiazol-2-yl)-2,5-Diphenyltetrazolium bromide] solution was prepared at 5 mg/ml in PBS. MMTV-PyMT cells were plated in 96 well plates in DMEM/F12 supplemented with 2% FBS, 20 ng/ml EGF, 10 µg/ml insulin and 1/10 vol of MTT solution was added to the medium. Cells were incubated for 4 hours at 37ºC, then media was removed and replaced with 200 µl of DMSO. The dye was completely dissolved by pipetting and the solution was incubated for 5 minutes at RT. Optical density was measured in a plate reader at 570 nm.

Supplemental File 5. Table 1: Primer Sets Used in This Study.

ID	Forward 5'-3'	Reverse 5'-3'
Rplp0	GATTCGGGATATGCTGTTGG	GTTCTGAGCTGGCACAGTGA
Krt18	CGAGGCACTCAAGGAAGAAC	AATCTGGGCTTCCAGACCTT
Tgfbr1	AAATTGCTCGACGCTGTTCT	CAACCGATGGATCAGAAGGT
Aldh1a3	GAGCGATCCTGGCTACTCTG	GACGAAAAAGGCATGAAGGA
Sox9	CAGCAAGACTCTGGGCAAG	TCCACGAAGGGTCTCTTCTC
Gata3	GCTACGGTGCAGAGGTATCC	AGAGATCCGTGCAGCAGAG
Mmp2	ACACTGGGACCTGTCACTCC	TGTCACTGTCCGCCAAATAA
Mmp9	CCAGATGATGGGAGAGAAGC	TTGAGGCCTTTGAAGGTTTG
Snai2	GAACCCACACATTGCCTTGT	GCAGAAGCGACATTCTGGAG
Zeb2	CTATTCCCCTGCATCAGCAT	GGCTTGTCAGTCCTTTCTCG
Vim	GCGAGGAGAGCAGGATTTCTC	GGGTGTCAACCAGAGGAAGT
Fn1	TGCACGTGTGTGGGGAACGG	CCCGGCCCTGACCAAAGCAG
Pdgfb	TGGTATCACTCCTGGAAGCC	AACAGAAGACAGCGAGGTGG
